# Muroid rodent phylogenetics: 900-species tree reveals increasing diversification rates

**DOI:** 10.1371/journal.pone.0183070

**Published:** 2017-08-16

**Authors:** Scott J. Steppan, John J. Schenk

**Affiliations:** 1 Department of Biological Science, Florida State University, Tallahassee, Florida, United States of America; 2 Department of Biology, Georgia Southern University, Statesboro, Georgia, United States of America; Tel Aviv University, ISRAEL

## Abstract

We combined new sequence data for more than 300 muroid rodent species with our previously published sequences for up to five nuclear and one mitochondrial genes to generate the most widely and densely sampled hypothesis of evolutionary relationships across Muroidea. An exhaustive screening procedure for publically available sequences was implemented to avoid the propagation of taxonomic errors that are common to supermatrix studies. The combined data set of carefully screened sequences derived from all available sequences on GenBank with our new data resulted in a robust maximum likelihood phylogeny for 900 of the approximately 1,620 muroids. Several regions that were equivocally resolved in previous studies are now more decisively resolved, and we estimated a chronogram using 28 fossil calibrations for the most integrated age and topological estimates to date. The results were used to update muroid classification and highlight questions needing additional data. We also compared the results of multigene supermatrix studies like this one with the principal published supertrees and concluded that the latter are unreliable for any comparative study in muroids. In addition, we explored diversification patterns as an explanation for why muroid rodents represent one of the most species-rich groups of mammals by detecting evidence for increasing net diversification rates through time across the muroid tree. We suggest the observation of increasing rates may be due to a combination of parallel increases in rate across clades and high average extinction rates. Five increased diversification-rate-shifts were inferred, suggesting that multiple, but perhaps not independent, events have led to the remarkable species diversity in the superfamily. Our results provide a phylogenetic framework for comparative studies that is not highly dependent upon the signal from any one gene.

## Introduction

The muroid rodents are arguably the most evolutionarily successful clade of mammals, with approximately 1620 species (30% of mammal diversity) that evolved from a single common ancestor approximately 25 million years ago (mya) [1]. Several evolutionary mechanisms are likely responsible for this diversity, which has led to at least four significant increases in diversification rates during its history [1] and perhaps as many as 24 [2]. Among these mechanisms, ecological opportunity in response to continental migration has been the best studied, but this process does not explain most increases in diversification rates [1], and certainly other mechanisms exist such as key innovations. Resolution of the muroid phylogeny is important to many areas of science because Muroidea contains many of the most common model organisms for experimental, ecological, and physiological research and comparative studies require well-resolved phylogenies. A resolved phylogeny is also critical to determining the processes that control diversification, and this diverse clade is an exceptional model system for such studies.

Although biologists are often interested in understanding diversification patterns among species-rich groups, conducting phylogenetic-based analyses on these groups is challenging because it is difficult to obtain a large number of tissue samples, the associated sequencing costs, and computational complexity. To date, analyses in muroids have focused on more manageable subgroups, similar to strategies in other diverse clades. However, this can lead to systematic biases in divergence analyses by ignoring the broader context and background diversification rate. Alternatively, one can sample sparsely among major groups, but this does not accurately depict full extant species diversity, nor does it provide good within clade branch lengths estimates. Supermatrix studies that combine data from multiple studies have been making progress in muroids, with increasing size, e.g., [1, 2]. An alternative is the set of supertree methods that combine and reconcile topological information from multiple published studies with overlapping taxon sampling to maximize taxonomic coverage. However, supertree methods discard the information-rich original data (usually DNA sequences), resulting in several criticisms that have been thoroughly debated elsewhere [3–5]. Mammalian supertrees [6–8] have been used as the framework for many hundreds of comparative studies and with muroids accounting for 30% of the species in those trees, assessing their accuracy is a critical endeavor.

With nearly a third of mammalian diversity represented by this relatively young group, much work has been conducted to better understand the diversification of Muroidea. Steppan et al. [9] identified four potential areas in the phylogeny where speciation rates accelerated, Fabre et al. [10] identified 24 shifts, and Schenk et al. [1] identified up to 20 shifts. Schenk et al. [1], and later Alhajeri et al. [11], identified the clade Oryzomyalia as resulting from ecological opportunity; however, it was less clear how and why the other clades with accelerated net speciation rates diversified at a higher rate than other muroids. It is also less clear how incomplete taxonomic sampling could influence diversification inference, and it is likely that our estimates will be more accurate as we approach complete sampling of extant species diversity. In addition to our primary goal of generating the most complete and robust phylogeny for this group to provide an improved framework for comparative and systematic studies, we reestimate diversification parameters and determine if they are robust to additional taxon sampling.

## Materials and methods

### Ethics statement

No new specimen collecting was conducted for this study and all tissue samples were loaned from accredited museums. Tissue samples were obtained from the following institutions: Field Museum of Natural History, Museum of Southwestern Biology, Carnegie Museum of Natural History, South Australian Museum, Texas A&M University, Royal Ontario Museum, Louisiana State University, Museum of Vertebrate Zoology, Berkeley, Texas Tech University, University of Kansas, American Museum of Natural History, United States National Museum, California Academy of Science, Sam Nobel Museum of Natural History, Burke Museum University of Washington, Museo de Historia Natural, Universidad Nacional Mayor de San Marcos, Peru, and museum-associated collectors.

### Taxonomic sampling

We included 904 ingroup taxa and nine taxa from Dipodoidea, the strongly supported sister-group to Muroidea [9]. We included 250 of the approximately 330 muroid genera. New sequences generated as part of this study were supplemented with sequence data from several sources, including samples from our previous studies [1, 9, 12–15] and GenBank. Candidate genes were assessed using the Phylota browser [16], choosing those with the widest coverage, emphasizing nuclear loci to minimize the issues with saturation that affect mitochondrial sequences at these depths of the phylogeny [13], but also including the most widely sequenced gene, mitochondrial cytochrome-b (*cytb*). All available sequences for these genes were downloaded using Phylota for subsequent screening (see below). All taxa and gene segments are listed in S1 Appendix.

### DNA sequencing

We sequenced five nuclear genes and one mitochondrial gene (*cytb*). Amplification and sequencing of exon 11 of breast cancer 1 (*BRCA1*), exon 10 of growth hormone receptor (*GHR*), exon 1 of interphotoreceptor retinoid binding protein (*Rbp3*), and the single exon of recombination activating gene 1 (*RAG1*) followed that of Schenk et al. [1]. Intron 2 and parts of exons 2 and 3 of acid phosphatase type V (*Acp5*) were amplified with AP5-564rev and AP5-120fwd [17], and sequenced with the primers AP5-139fwd and AP5-545rev [17], or the amplifying primers for some sequences. *Cytb* was amplified with L14725M and H15915V [18], L14725M and S199 (5’–CCTCARAATGATATTTGTCCTCA), or primers designed here, S330 (5’–CCAATGACATGAAAAATCATCG) and S331 (5’–GGGGATAGTCCTTCCTTCTTG). The S330/S331 primer pair was optimized for Sigmodontinae, but also amplified other muroids.

Amplification followed that of Schenk et al. [1] for *GHR*, *Rbp3*, and *RAG1*. PCR conditions for all genes included an initial denature period of 94°C for 2 min, followed by 35–40 cycles of 94°C for 30–45 seconds, 54–62°C for 30–60 seconds, and 72°C for 1.5 minutes, and the reaction was terminated with a single cycle of 72°C for 6–7 minutes. The *Acp5* annealing temperature was optimized at 62°C for 40 sec, whereas *cytb* was optimized at 55°C for 45 sec for the primer pair S330/S331, and 54°C for 1 min for the primer pairs L14725M/H15915V and L14725M/S199.

Fresh tissues were not available for *Ichthyomys stolzmanni* and so we amplified *Rbp3* from dried skin in 100–200 bp fragments using primer pairs from Jansa and Weksler [19], cloned, and then sequenced. The amplicons were inserted into Topo TA vectors (Life Technologies Corporation, Grand Island, NY, USA), which were transformed into competent cells and raised on agar plates. Vectors were then isolated from the cells and sequenced with M13 forward and reverse primers. Preliminary phylogenetic analyses were conducted on the individual clones separately to test for possible contamination before they were combined into a single sequence. Gaps between the clones (if any) were made continuous by denoting the nucleotides as ambiguous (N). Newly derived sequences were accessioned with GenBank under accession numbers KY753930-KY754183, MF074854-MF074869, MF074873-MF074944, MF097704-MF097815, MF097816-MF097959, and MF110300-MF110573 (S1 Appendix).

### Screening procedures for publically available sequences

In our previous work [1], our detailed taxonomic sampling revealed evidence for numerous misidentified, chimeric, or error-containing sequences deposited on GenBank. We therefore developed and implemented an exhaustive screening procedure before using published sequences. All muroid sequences available on GenBank for each gene were downloaded, aligned with all sequences generated in our lab, and subjected to multiple iterations of phylogenetic analyses. Initial alignments ranged in extent from >6,000 sequences for *cytb* to those for which all available sequences were generated by our lab group (> 400 for *RAG1*, 166 for *BRCA1*). When multiple sequences were available per species, preference was given to sequences meeting the following criteria: (1) from labs or researchers associated with museums or with extensive personal experience with the species in question, (2) published in a peer reviewed journal, (3) from vouchers deposited in museums [20], (4) from within the generally accepted range of the species (IUCN) [21], (5) with longer sequence, (6) containing no unexpected stop codons, gaps, or atypically large number of amino acid substitutions relative to the respective marker, (7) were concordant topologically with evidence from other genes, (8) were part of monophyletic groups with putative conspecifics, indicating corroboration for species identification, and (9), were derived from the same vouchers that were the source for other gene seqeunces. We excluded GenBank accessions if the data were unpublished in most cases. All iterations involved comparing trees among individual genes to detect any discordance that might indicate a misidentification. In cases where species were represented by multiple individuals, after consideration of the aforementioned preferences, we randomly chose one accession if they formed a monophyletic group and they were all the same sequence length. When species did not form monophyletic groups, we first verified the determination in museum records (if possible) and their associated publications, because some vouchers had species identifications reassessed by the lending museums subsequent to their being loaned to the publishing labs, and we then updated names accordingly. Some species appeared polyphyletic and had one or more individuals that were recovered in an unexpected relationship relative to other conspecific individuals in preliminary analyses. We removed the accessions with unexpected placement from analyses if their discordance was great enough (e.g., different genus, tribe, subfamily) and appeared to be due to contamination or lab error. If a species was non-monophyletic and the sequences were from experienced labs but different localities, we provisionally attributed non-monophyly to the discovery of possible cryptic species or true paraphyly in a widespread species, and consequently added geographic identifiers to the names, retaining the sequences. For species that were minimally paraphyletic and did not include widely separated localities, we randomly sampled a single accession to represent the species unless the above criteria allowed us to rank the samples. In most cases, these paraphyletic species contained another species that was expected to be closely related based on prior work (e.g., morphology). The few cases where we excluded multiple lineages within a species-complex might result in a less-accurate depiction of species boundaries, but should not bias our results in any substantial way [21]. We argue that protocols such as this are critical to prevent the perpetuation of erroneous results through the literature. Some relevant examples are discussed further in the Discussion.

The problem of nonmonophyly was especially pronounced in the *cytb* data, and especially with the most intensely studied genera (in terms of numbers of labs) exhibiting the greatest proportion of non-monophyletic species. The most problematic genera were the sigmodontine *Oligoryzomys* (long-tailed rice rats; all eight species with multiple samples appeared polyphyletic), the neotomine deer mice *Peromyscus*, and the murines *Rattus* and *Niviventer*. We attribute most of the discordance to differing understanding of diagnostic traits of species among multiple labs. Pseudogenes were detected in several *cytb* sequences based on the presence of stop codons, and these sequences were removed from the data matrices.

We retained some sequences from GenBank about which we had doubts regarding species level status and synonymy, based on short branch lengths separating possible conspecifics (e.g., species according to Musser and Carleton, [21]). We retained the published names in part to stimulate reexamination of these taxa.

### Phylogenetic analysis

We inferred phylogenetic relationships among muroid species with maximum likelihood (ML) and Bayesian inference (BI) approaches. A best-fit model was chosen by selecting one of 57 DNA substitution models that fit the data best while avoiding overparameterization with the Akaike information criterion [22]. Model selection was conducted on the gene data sets individually, and on the concatenated data using ModelTest 3.7 [23]. These models were applied with fixed parameters in ML analyses, and the models, or the next available, more parameterized model was applied in BI analyses.

Phylogenetic inferences were optimized with ML in RAxML [24]. Four-hundred replicated searches were conducted on the Cipres Science Gateway [25], with eight batches of 50 replicated searches, each with unique, randomly chosen seed value for the initial parsimony tree. The sequence data were partitioned by codon position for single protein coding genes. Concatenated data were partitioned by codon position in exons, by introns, and by codon position in the mitochondrial *cytb* gene. The concatenated and *cytb* RAxML ML trees were subjected to additional phylogenetic analyses as the starting tree in an unpartitioned ML analysis in PAUP [26]. Given the large amount of data in these data sets, we searched tree space with the tree-bisection-reconnection algorithm for a limited time of 300 hours.

We used nonparametric bootstrap (BS) proportions to infer the level of support for clades. We applied the rapid bootstrapping criterion on individual gene data sets and the concatenated data set with RAxML on the Cipres Science Gateway. We conducted 1000 BS replicates and summarized the results on the best ML tree estimated in the separate RAxML analyses outlined above.

Bayesian inference was conducted in MrBayes 3.2.1 [27]. For the individual gene data sets, we applied a flat Dirichlet prior on partitioned data, where each partition was optimized individually with the GTR+I+Γ DNA substitution model. We ran analyses on each dataset for 4 × 10^7^ generations, sampling every 1 × 10^3^ generations. In the concatenated data set, we applied several partitioning schemes and tested their fit to the data with Bayes factors. Bayes factors were estimated from harmonic likelihood scores estimated with the stepping stone model. Multiple analyses with varying degrees of simplified partitioning schemes of up to 4.4 × 10^7^ generations were run, lasting several months each, but none reached convergence (standard deviation of split frequencies > 0.38) despite simplifying partitioning schemes. In contrast to the individual-gene analyses that were highly concordant with ML results, all concatenated BI analyses had some anomalous clades. Bayesian approaches, while having the strength of estimating uncertainty, can do a poor job sampling parameter and tree space when the number of parameters is high, whereas ML is only searching tree space. It appears that a combination of the large size of this dataset and some isolated cases of species not sharing genes (e.g., *Microtus majori* with *M*. *mongolicus/M*. *xanthognathus*) precluded efficient Bayesian analysis and we do not report those results further.

Species were represented by multiple genes in most cases, including 840 species for *cytb*, 576 for *Rbp3*, 438 for *GHR*, 387 for *RAG1*, 375 for *AP5*, and 165 for *BRCA1*. In total, we included 2,781 sequences from 913 taxa, 904 of which were from Muroidea, with a mean of three genes and 3,070 bp per species (median 2,581 bp). The matrix was 35% complete. Individual ML gene trees are available in Newick format S1–S6 Files, and as pdf S1–S6 Figs.

### Divergence time analysis

Because Bayesian dating analysis using BEAST [28] showed little evidence of converging even after a series of multi-month long runs (see below), we applied penalized likelihood in r8s v.1.81 [29] to estimate a chronogram. We calibrated the phylogeny with 28 fossil constraints (Table 1), including the 14 calibration points from Schenk et al. [1]. Criteria for the confidence intervals for the 14 new calibrations can be found in Table 1. Minimum and maximum ages corresponded to the 95% confidence limits from the Marshall index [30] as calculated by Paleobiology Database [31] (PBDB; accessed June 29, 2016), except when there was a large and significant correlation between rank age and gap size, in which case we used the 90% interval using the method of Solow [32]. For Dipodoidea, we used the PBDB estimated maximum age of 62.3 mya rather than the more conservative and arbitrary 70 mya from Schenk et al. [1]. These calibrations were analyzed with the fossil cross-validation procedure in r8s [33, 34] to test for consistency among ages. Cross validation was conducted on the ML phylogram using a range of smoothing parameters from 1 to 10,000. A smoothing parameter value of 100 was found to minimize deviations among calibrations, was most consistent visually with relative branch lengths in the ML phylogram, and was the value used in the final analysis. A second approach to validating calibrations applied only the calibration prior information in a BEAST analysis that was run for 1 × 10^7^ generations without sequence data, and we expected that if the priors were not interacting with one another, the posterior distribution should be similar to the prior distribution. Both the partitioned ML tree and the chronogram are available in Newick format from TreeBase (accession 20819).

**Table 1:** Fossil calibrations used for dating analyses.

Node #	Taxon	MinAge	MaxAge	Log-StDev	Offset
1	Dipodoidea^a^	48.6	62.3	1.928	46.16
2	Rhizomyinae^a^	23	30.03	1.198	22.86
3	*Reithrodontomys*^a^^,^ ^b^	1.8	6.89	1.076	1.630
4	*Onychomys*^a^^,^ ^b^	4.9	10.28	1.169	4.753
5	Sigmodontini^a^	4.9	14.93	1.408	4.801
6	*Holochilus*^a^^,^ ^b^	0.8	1.24	0.140	0.006
7	*Reithrodon*^a^	3.5	—	0.180	2.756
8	*Necromys*^a^	3.5	4.625	0.326	2.915
9	*Auliscomys*^a^	4	6.8	0.692	3.679
10	*Acomys/Deomys*^a^	5.3	8.89	1.927	5.258
11	Gerbillinae/Deomyinae^a^	16	23.7	1.251	15.868
12	Murinae^a^	12.1	14.05	0.885	9.767
13	*Apodemus*^a^	5.3	7.2	0.515	4.871
14	*Neotoma*	5.3	10.3	1.001	5.113
15	*Lemmus*	2.6	3.2	0.185	1.860
16	*Ondatra*	1.86	2.83	0.211	1.156
17	*Mesocricetus/Cricetulus*	4.9	5.3	0.150	4.049
18	*Mastomys*	2.6	4.3	0.311	2.000
19	*Mus*	5.3	7.2	0.515	4.871
20	*Rhabdomys*	2.6	8.2	0.674	2.270
21	*Dasymys*^b^	2.6	4.8	0.674	2.270
22	*Aethomys*	3.6	5.67	0.470	3.138
23	*Otomys*	2.6	5.3	0.674	2.270
24	*Dendromus*	5.3	7.2	0.515	4.871
25	*Mystromys*	3.6	5.3	0.470	3.138
26	*Meriones*^a^^,^ ^b^	2.6	8.72	0.674	2.270
27	*Myodes*^a^^,^ ^b^	2.6	5.87	0.181	1.857
28	*Arvicolinae*^a^^,^ ^b^	4.9	9.0	—	—

MinAge and MaxAge are the minimum and maximum ages, respectively, applied in r8s and correspond to the 95% credibility intervals in BEAST.

^a^Calibrations used in Schenk et al (2013).

^b^Solow 90% index.

We also applied the uncorrelated lognormal relaxed clock method in BEAST v1.8.0 [28] to estimate divergence times. The means for all calibrations were 0 and the standard-deviation and offset for each calibration are reported in Table 1. The concatenated data were partitioned by introns and by codon position in exons the mitochondrial DNA as in the ML and BI analyses. The GTR+I+Γ model was applied to all partitions and parameters were unlinked. To greatly speed up the analysis, we supplied the best ML tree estimated from the concatenated data in RAxML as a starting tree. We ran two replicates with Dipodoidea as the outgroup for 6 × 10^7^ generations, and sampled every 2 × 10^3^ generations. Convergence between the two analyses was assessed in AWTY [35]. Effective sample sizes and plots of -lnL scores were examined in Tracer [36] to assess whether the chain sampled appropriately. Given the difficulties in reaching convergence on such a large data set, we adjusted operators of the DNA substitution model on the basis of preliminary results to conduct a more efficient BEAST analysis. Dates estimated by r8s are reported in the Results below and the chronogram from the Beast analyses is available from S7 File. As described above, because of the difficulty in obtaining stationarity, we favor the results of the r8s analysis, using them in diversification analyses, and report the BEAST results only to corroborate ages estimated in r8s.

### Diversification rates

The rates at which lineages diversified across Muroidea were explored with several approaches. We plotted the log number of lineages through time with lineage through time plots with the Ape package [37] in R. We also plotted new lineage accumulation rates with a sliding window analysis from Meredith et al. [38], that we coded in R (available: https://github.com/johnjschenk/Rcode/blob/master/SlidingWindow.R). The sliding window analysis was conducted across the muroid chronogram with a 2-million-year window.

Uncertainty in the number and locations of diversification rate shifts have been identified in previous studies [39–41] and was directly assessed by implementing the Bayesian analysis of macroevolutionary mixtures (BAMM) method [42]. We used BAMM v2.5.0 to determine the most likely number of rate diversification shifts. Concerns have been raised about possible pathologies in BAMM likelihood calculations [43], but those concerns were addressed in the current version of the software and may no longer apply. We sampled every 2000 generations for 10^7^ generations with the speciation-extinction model and a prior block chosen with BAMMtools in R. Incomplete sampling of muroids was accounted for by incorporating a sampling frequency of 0.69461. The BAMMTools package was used to process and view the results.

## Results and discussion

### Phylogenetics: Relationships among subfamilies

**Fig 1:**
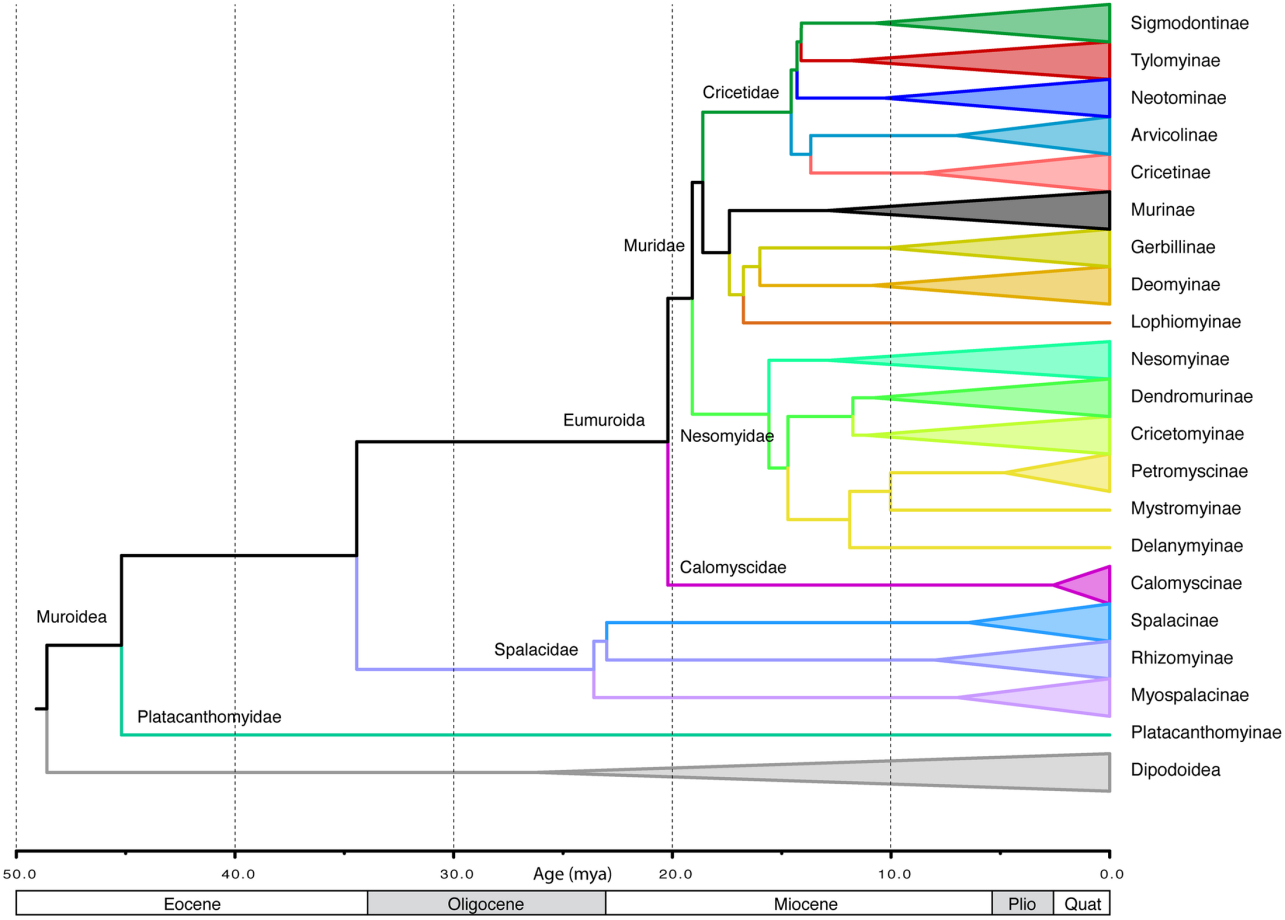
Backbone summary phylogeny. Maximum-likelihood chronogram summarizing the relationships among subfamilies. Each polytypic subfamily has its diversity represented by equal-width cones; the depth of each corresponds to the most recent common ancestor of the crown clade. Clades are colored according to the classification of subfamilies used in the text.

**Fig 2:**
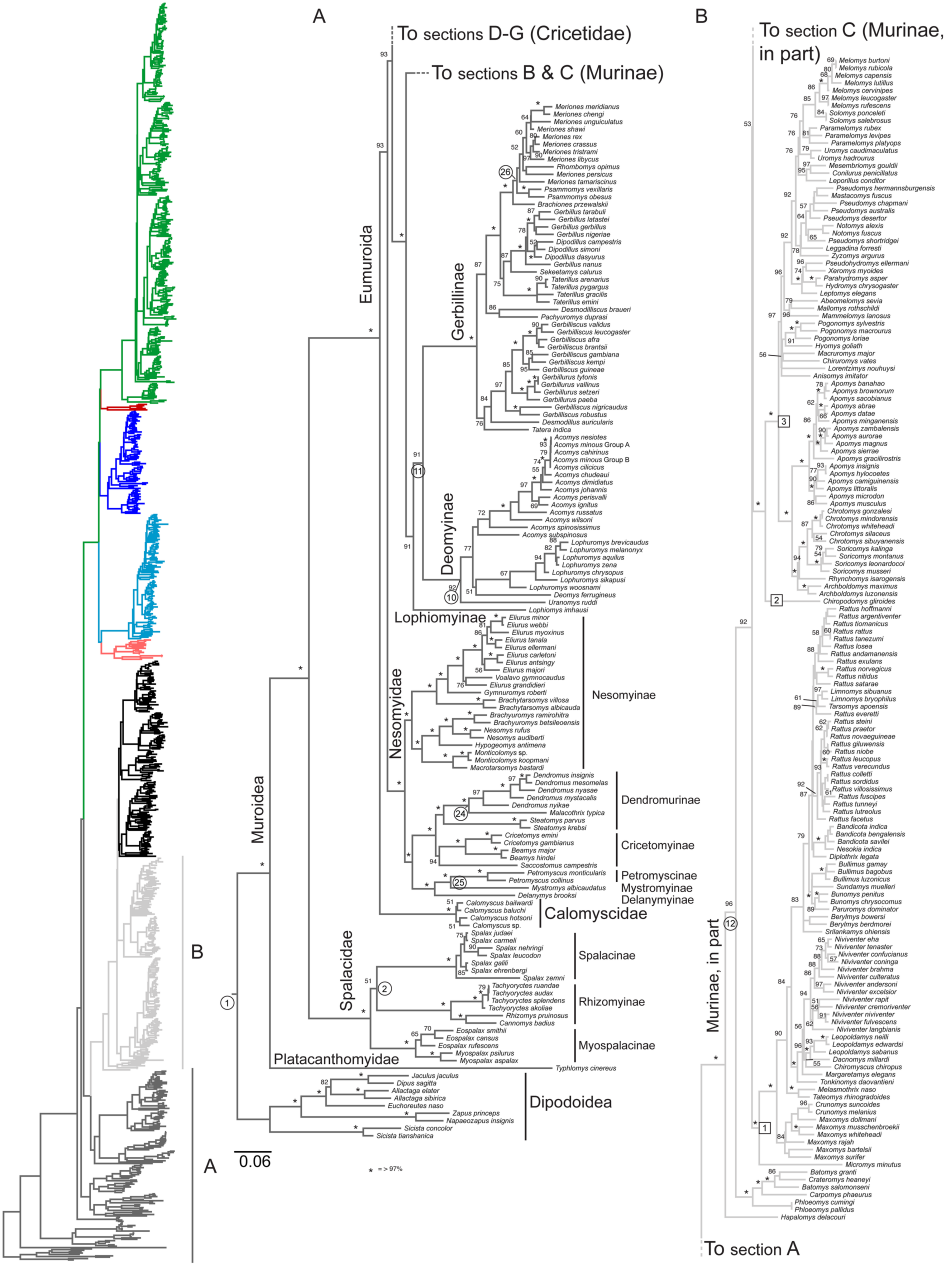
Basal region of full 900-species phylogeny, Platacanthomyidae, Spalacidae, Calomyscinae, Nesomyidae, and part of Muridae. Colored ML phylogram on left is the entire tree, color coded by subfamily as in Fig 1 for non-highlighted sections of the tree. Section A (containing the outgroup, Platacanthomyidae, Spalacidae, Nesomyidae, Calomyscinae, and all of Muridae excluding Murinae) is highlighted in dark grey and expanded in detail to right; Section B (containing part of Murinae) is highlighted in light grey. Numbers above branches are the ML bootstrap values; “*” indicates 98–100%, values below 50% not shown. Boxed numbers are tribal-level clades discussed in the text. Circled numbers are the calibration nodes, numbers as in Table 1. Scale bar indicates expected amount of change along branches.

**Fig 3:**
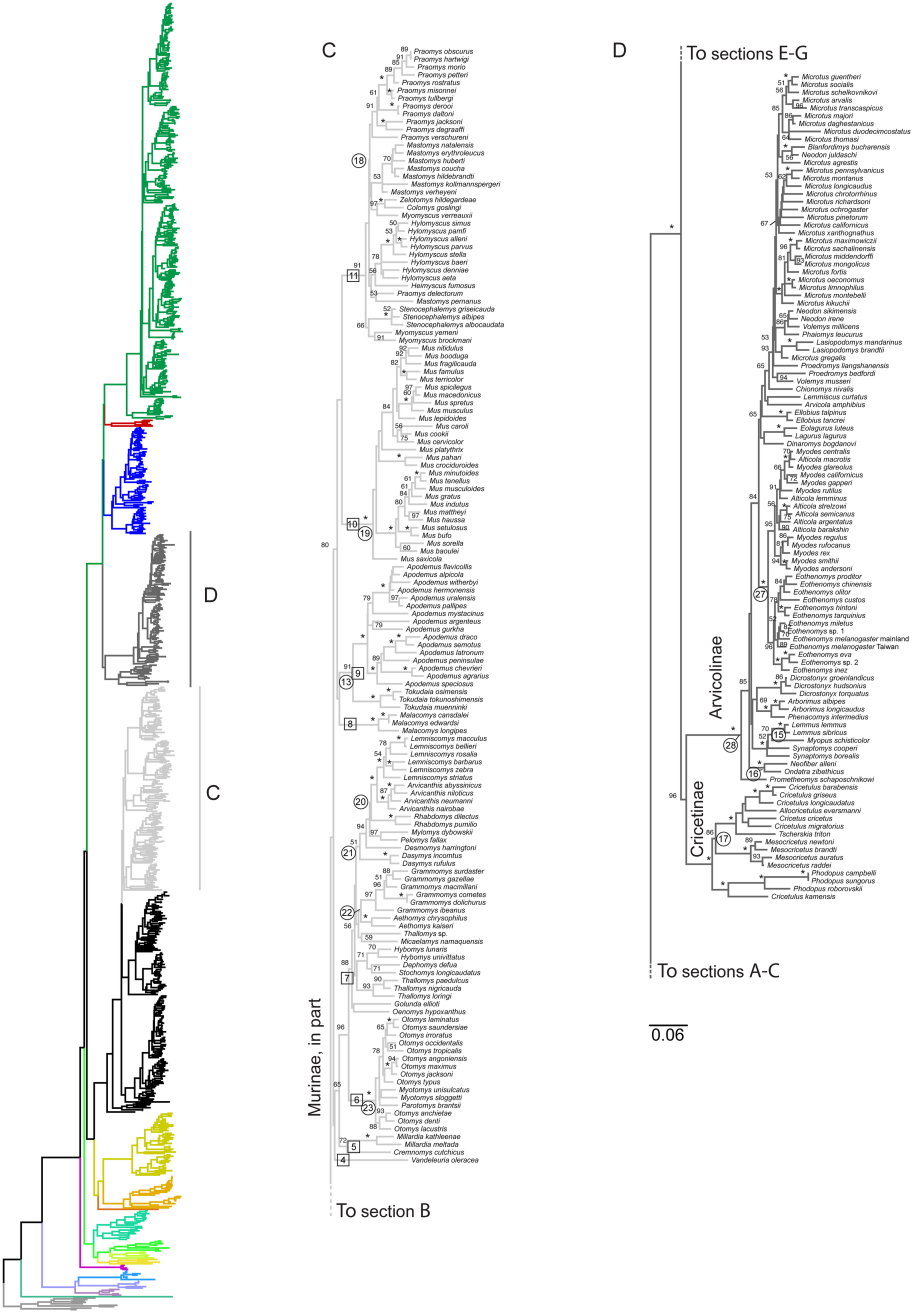
Intermediate region of full 900-species phylogeny, Murinae (in part, African and Indian clades) and Cricetidae (in part, hamsters Cricetinae and voles Arvicolinae). Colored ML phylogram on left is the entire tree, color coded as in Fig 1 for non-highlighted sections of the tree. Section C (containing the African and Indian murine genera and their descendants) is highlighted in light grey and expanded in detail to right; Section D (containing Cricetinae and Arvicolinae) is highlighted in dark grey. Numbers above branches are the ML bootstrap values; “*” indicates 98–100%, values below 50% are not shown. Boxed numbers are tribal-level clades discussed in the text. Circled numbers are the calibration nodes, numbers as in Table 1. Scale bar indicates expected amount of change along branches.

**Fig 4:**
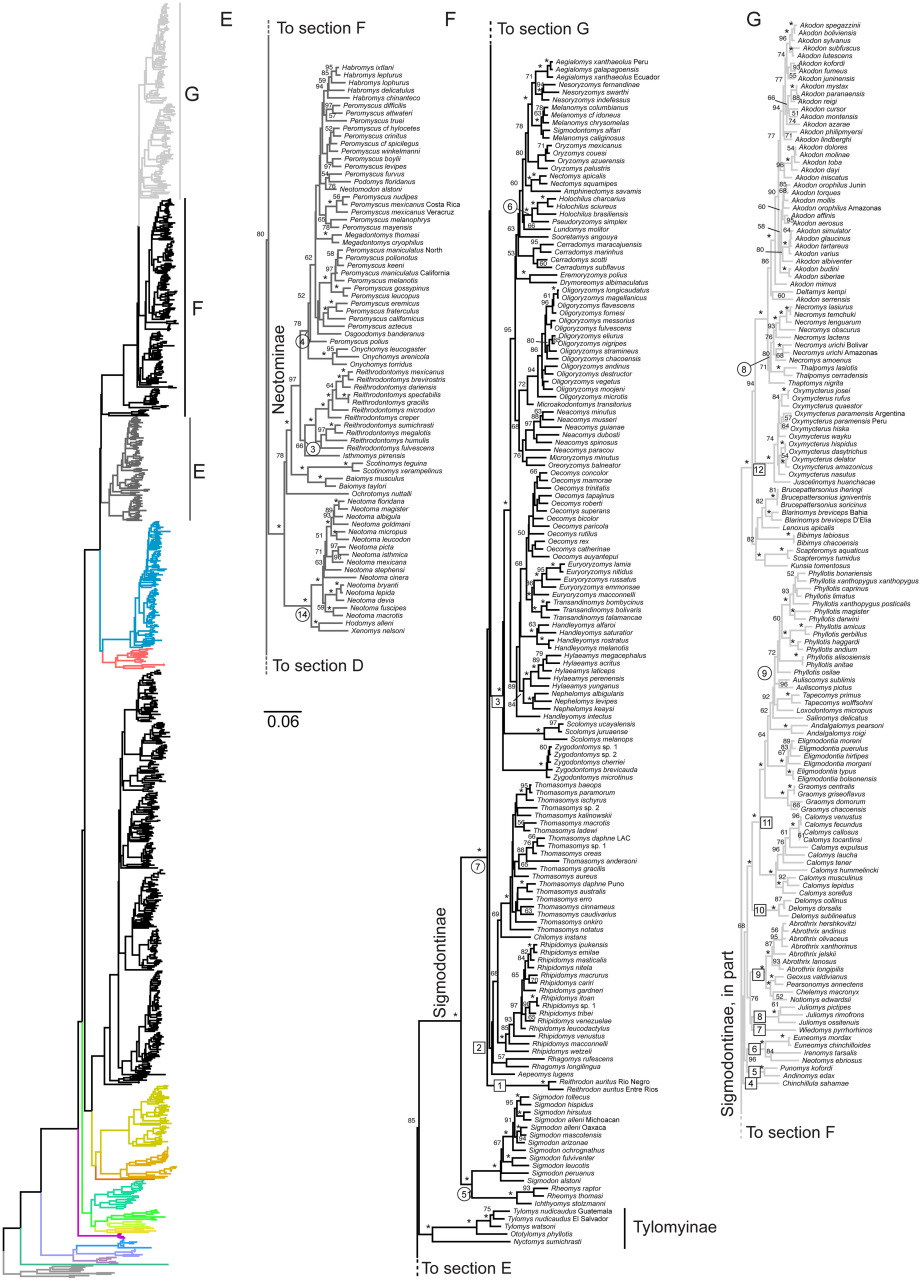
Terminal region of full 900-species phylogeny, New World Cricetidae. Colored ML phylogram on left is the entire tree, color coded as in Fig 1 for non-highlighted sections of the tree. Section E, Neotominae, is highlighted in dark grey and expanded in detail to right; Section F (containing Tylomyinae and a portion of Sigmodontinae) is highlighted in black; Section G (containing the remainder of Sigmodontinae) is highlighted in light grey and expanded to the right. Numbers above branches are the ML bootstrap values; “*” indicates 98–100%, values below 50% not shown. Boxed numbers are tribal-level clades discussed in the text. Circled numbers are the calibration nodes, numbers as in Table 1. Scale bar indicates expected amount of change along branches.

**Fig 5:**
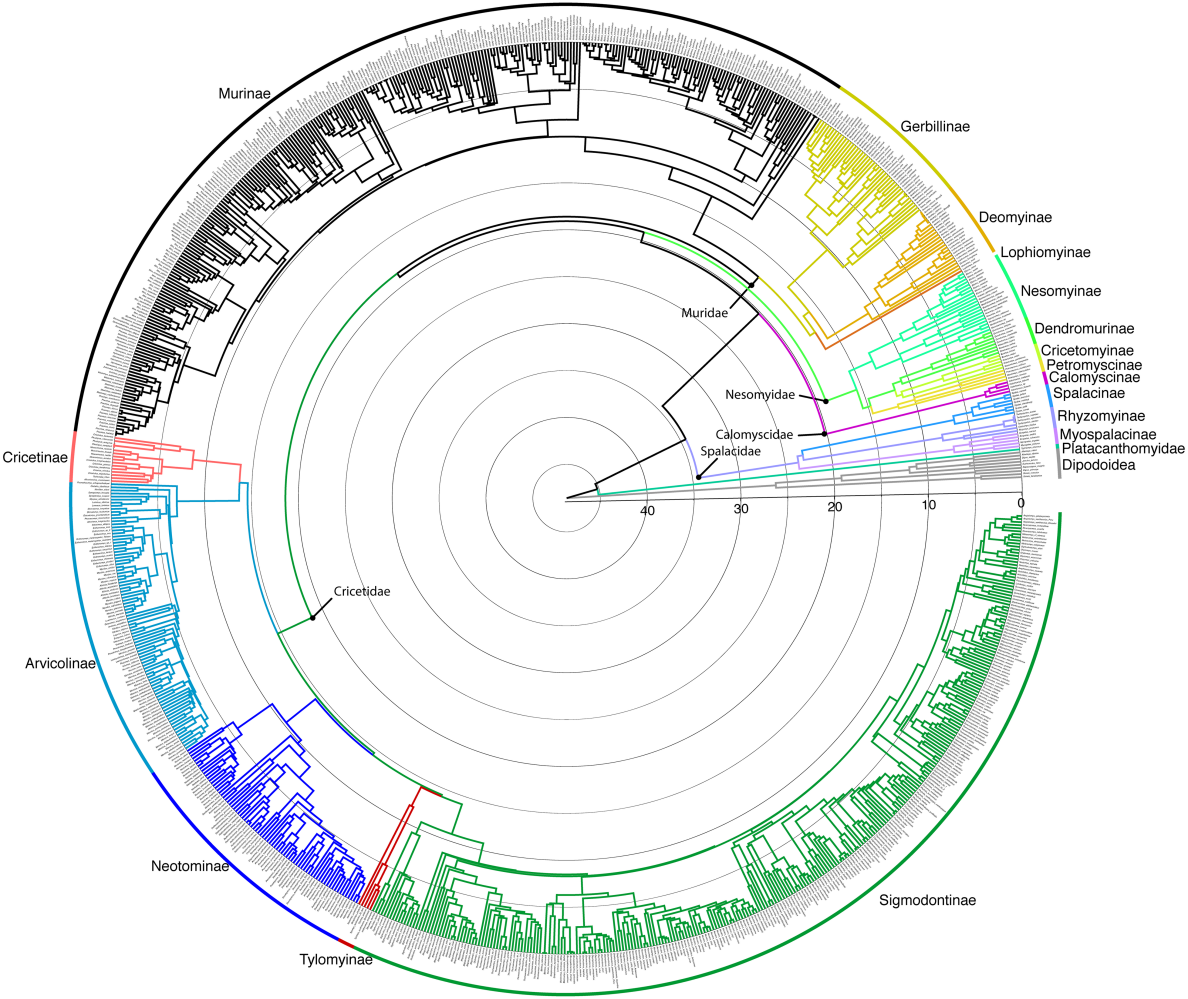
Chronogram of Muroidea. Colored chronogram estimated using penalized-likelihood on the ML tree, color coded as in Fig 1. Time scale in million years ago.

The GTR+I+Γ DNA substitution model best fit the individual gene data sets, except for *BRCA1*, in which the GTR+Γ model fit best. This model was applied in RAxML analyses, which found a single most likely tree for all data sets. No significant conflicts in topology among the gene trees were identified, and the discordance that did exist was limited to rearrangements involving very short branches, mostly towards the tips. Most nodes were fully concordant across all genes (S1–S6 Files and S1–S6 Figs). The ML tree resulting from analyses of the concatenated dataset was fully resolved and robustly supported (Figs 1–4). All deep and medium regions of the tree are concordant with Schenk et al. [1] and most recent nuclear DNA phylogenies, and the backbone tree summarizing subfamily relationship is shown in Fig 1. Muroids exhibited sequential divergences of the depauperate Platacanthomyidae (containing two monotypic genera, only one of which has genetic data), then the fossorial Spalacidae, followed by Eumuroida. The basal split between the Platacanthomyidae (*Typhlomys*) and all remaining muroids is estimated to have occurred around 45.2 mya in the early Eocene (Fig 5). Spalacinae and Rhizomyinae appear to be sister taxa, but with weak support (51% BS). The crown groups of each of the three spalacid subfamilies appear to be relatively recent radiations, helping to account for their long being recognized as distinct taxa. Eumuroida then diversified rapidly into four families starting around 20.2 mya in the early Miocene (Fig 5). Basal branches within Eumuroida are short, but data are sufficient to resolve relationships among the four constituent families with strong support (93% BS for both nodes). The monogeneric Calomyscidae (the “mouse-like hamsters” of western Asia) are sister to all other Eumuroida, with the exclusively African Nesomyidae sister to the clade consisting of the Old World Muridae and the Holarctic/Neotropical Cricetidae. The Muridae plus Cricetidae clade contains approximately 94% of muroid diversity and 27% of all mammals. Below we describe the major phylogenetic results. Detailed discussions within some subfamilies are too extensive to be covered here, and are addressed in more focused publications elsewhere (e.g., Gerbillinae and Deomyinae,[44]; Neotominae Miller et al., in prep.; Arvicolinae Conroy et al., in prep; Sigmodontinae, Steppan et al., in prep).

The molecular data are in general agreement with paleontological cladistics of the fossorial Spalacidae. The splits among the three spalacid subfamilies occurred quickly, within less than 1 million years, starting around 23.6 mya. These dates are compatible with the fossil record, with the earliest member of the family, *Prokanisomys* dated at around 24 mya by Lopez-Antonanzas & Flynn [45], although Paleobiology Database indicates greater uncertainty regarding the stratigraphic dating, yielding a range of 16–23 mya [31]. Within the Rhizomyinae, the bamboo rat *Cannomys* and Asian mole-rats *Rhizomys* are sister taxa that diverged about 6.0 mya, and together they split from the African mole-rats *Tachyoryctes* about 8.0 mya. Both of these times are compatible with the fossil record, with the former split estimated around 2.5 mya—provided extant *Rhizomys* are closer to *Cannomys* than to the extinct *R*. *shansius*, or 4.5–10 mya if they are not [45]. The split among all three genera was estimated by Lopez-Antonanzas & Flynn [45] at between 7.5 and 10 mya, depending on the resolution among species of the extinct *Miorhizomys*.

### Phylogenetics: Nesomyidae

Within the morphologically diverse Nesomyidae (Fig 2, section A), the basal split was between the Malagasy endemic radiation Nesomyinae—a clade whose members have at times been distributed across as many as four subfamilies [21]—and the remaining three to five subfamilies originated around 17.4 mya. Monophyly is corroborated for the climbing mice Dendromurinae (although we lack the monotypic genera *Dendroprionomys*, *Megadendromus*, and *Prionomys*) and the Cricetomyinae. The pouched-mice *Saccostomus* appear to be members of the Cricetomyinae, as conventionally understood, in contrast to Jansa et al. [46] who considered them members of Dendromurinae based on *Rbp3* and *cytb* (support for this arrangement was noted to be weak by Jansa and Weksler [19]). However, the position of *Saccostomus* is inconsistent among genes, being either in Cricetomyinae (*BRCA1*, *RAG1*), Dendromurinae (*Acp5*, *cytb*), sister to both (*GHR*), or mixed in a paraphyletic Dendromurinae along with the other cricetomyines (*Rbp3*), reflecting the short branches connecting the basal members of these two subfamilies. Each polytypic genus within these subfamilies is also monophyletic (hamster-rats *Beamys*, giant pouched-rat *Cricetomys*, climbing-mice *Dendromus*, fat-mice *Steatomys*). Musser and Carleton [21] separated the two genera forming Petromyscinae—*Delanymys* and the rock-mice *Petromyscus*—into separate subfamilies because the genera shared few if any defining characteristics and were morphologically divergent [47]. We choose to reunite them here, and include the southern African white-tailed mouse *Mystromys* (Mystromyinae) as well, because this expanded clade is comparable in age to all other recognized subfamilies (stem age of 14.7 mya, compared to a range of 11.7–23.6 mya for all others), while splitting into three subfamilies would make their divergences atypically recent (stem ages 10.0–11.1 mya). Petromyscinae Roberts 1951 is the oldest available family-level name for this clade. Dental morphology also supports this clade [48]. Both options are consistent with the phylogeny; the only other option in conflict would be to include *Delanymys* and *Petromyscus* in a traditional but paraphyletic Petromyscinae to the exclusion of *Mystromys*, which we do not advocate.

We have increased the sampling within nesomyine genera (especially the tufted-tail rats *Eliurus*) over previous studies that included nuclear genes [19, 49], allowing more complete tests of monophyly. Paraphyly of *Eliurus* is supported by *cytb* and *Rbp3* with respect to *Voalavo* (*E*. *grandidieri* is either sister to or basal to *Voalavo*, with a very short branch separating the three clades in both genes), supporting the removal of *E*. *grandidieri* from *Eliurus*. We date the crown-group diversification to around 12.8 mya and the colonization of Madagascar to between that date and 15.6 mya, more recent than the 18–30 mya estimates from Poux et al. [50].

### Phylogenetics: Muridae

Muridae and Cricetidae are the first and second largest families of mammals, respectively, and are well supported as sister groups. Within Muridae (Fig 2, Section A), the murines are sister to the clade consisting of the monotypic giant African maned rat *Lophiomys* plus Gerbillinae and Deomyinae, with the Gerbillinae (gerbils) sister to the Deomyinae (spiny mice and relatives). Divergence dates are approximately 17.4 mya and 16 mya, respectively. Relationships within these latter two subfamilies are consistent with those found by Alhajeri et al. [44] who had several additional species and loci. In particular, the primary traditional tribes and divisions within Gerbillinae are all paraphyletic, as are several of the larger genera. Our results are generally concordant with those of Chevret and Dobigny [51] who noted that a major systematic revision is needed. Regarding Deomyinae, a subfamily only relatively recently recognized as monophyletic [52], there is consistent although not strong support for *Acomys* (spiny mice) to be sister to the link-rat *Deomys* plus brush-furred mice *Lophuromys*, with *Uranomys* sister to the three other genera.

Within the Old World mice and rats Murinae (Fig 2, section B), we identified the marmoset mice *Hapalomys* of SE Asia lineage diverging from remaining Murinae around 12.9 mya. This genus of two species has been poorly studied before and enigmatic, with “highly derived” dentition [21], and our results corroborate the only other study to include it [53]. Notably, *Hapalomys* had never been suggested as being so divergent from any other murine. Morphological traits suggested a relationship with the pencil-tailed tree mouse *Chiropodomys* (Musser and Newcomb, 1983). *Hapalomys* had been placed in the Micromys Division by Musser and Carleton [21]. Molecular data now reveal that division to be polyphyletic [1, 53, 54], a collection of early diverging lineages that collectively form a grade that is not closely related to any other group nor each other, and included *Chiropodomys* (sister to Hydromyini), Eurasian harvest mouse *Micromys* (sister to Rattini), long-tailed climbing mice *Vandeleuria* (sister to Arvicanthini plus Millardini), and the unsampled pygmy tree mice *Haeromys* and red climbing mouse *Vernaya*. Previously the earliest divergent murine clade revealed by molecular phylogenies [1] had been the Philippine cloud rats and cloud runners (Phloeomyini; *Phloeomys*, *Batomys*, and *Carpomys*).

Excluding the *Hapalomys* and Phloeomyini clades, we recovered a very rapid diversification of primarily Eurasian clades with one or two African lineages that is poorly resolved, the “core Murinae” [9]. Following the classification scheme of Lecompte et al. [55] as a template, there are 11 tribal-level lineages arising from this near-polytomy (boxed numbers in Fig 2, Section B, and Fig 3, Section C): (1) Rattini, (2) *Chiropodomys*, (3) Hydromyini, (4) *Vandeleuria*, (5) Millardini, (6) Otomyini, (7) Arvicanthini, (8) Malacomyini, (9) Apodemini, (10) Murini, and (11) Praomyini. This diversification spread over an estimated 1.8 my (10.1–8.3 mya). Only two of the 29 divisions of Musser and Carleton [21] were not sampled here: the monogeneric *Echiothrix* and *Hadromys* divisions. Rowe et al. [54] showed that the spiny-rats *Echiothrix* fall within the Rattini with other shrew and water rats of Sulawesi. The poorly known Pithecheir Division of mainland and insular SE Asia is only represented here by *Margaretamys*, but it also falls well nested within Rattini. Cumulative molecular evidence shows the Pithecheir Division, like the Micromys division, to be polyphyletic. *Pithecheir* belongs in the Millardini [53] and *Lenothrix* joins *Margaretamys* in the Rattini [53].

Our sampling within the diverse Rattini is one of the most extensive to date, and reinforces prior evidence of the paraphyly of scientifically and ecologically important *Rattus*. *Rattus facetus* is well supported (92% BS) as the sister-group to the Sahulian (Australia/New Guinea) species; the *R*. *leucopus* (New Guinea) and *R*. *fuscipes* (Australian) species groups of *Rattus*. In addition, the Philippine endemics *Limnomys* (mountain rats) and *Tarsomys* (long-footed rats) were most closely related to the fellow Philippine endemic *R*. *everetti* (89% BS). The results slightly modify the contents of two diverse and taxonomically important divisions within Rattini proposed by Musser and Carleton [21]; the Rattus Division (*Rattus* through *Berylmys*) is supported but with the basal inclusion of *Srilankamys* from the Dacnomys Division (83% BS), while the Dacnomys Division includes *Margaretamys* of the Pithecheir Division plus the more recently described *Tonkinomys* (100% BS). Although not all basal core murine nodes were strongly supported, reconstructing geography on the optimal tree yielded the following insights. Two of the major lineages that form Section B (Fig 2) are all SE Asian, eastern Asian, or Sahulian, with the exception of *Micromys* that is widespread in Eurasia. Then, within Section C (Fig 3), the basal lineages are predominantly Indian (*Vandeleuria*, Millardini, basal *Mus saxicola*, and *Golunda*) or African (Otomyini, Arvicanthini, Malacomyini, some *Mus*, and Praomyini). The colonizations of Africa appear to have occurred around 9.7 mya (Figs 3 and 5).

Hydromyini (murine lineage 3) consisted of two major clades, the Philippine endemic radiation of shrew mice (*Apomys*, *Archboldomys*, *Chrotomys*, *Rhynchomys*, and *Soricomys*), containing over 32 species in a small area [56], and the morphologically diverse Sahulian radiation that diversified first in New Guinea around 6 mya (e.g., *Anisomys*, *Hyomys*, *Mallomys*, and the large, semiaquatic water rat *Hydromys*) and then continued 1.5–2.0 million years later in Australia (e.g., forest mouse *Pseudomys* and the bipedal hopping-mice *Notomys*). Lecompte et al. [55] resurrected Hydromyini to contain this well supported clade, but a name that had been previously applied to just taxa with convergently reduced, basin-shaped molars, including the giant water rats *Hydromys* (a large bodied, semi-aquatic piscivore and crustaceavore of New Guinea and northern Queensland) and relatives in New Guinea along with the shrew-mice *Chrotomys* [57, 58] of the Philippines. The two radiations forming this tribe are geographically and morphologically very distinct; even the dentally convergent taxa have distinctly different lifestyles, locomotion, and diets. If the tribal name Hydromyini is retained, it would be beneficial to recognize the clades formally because of their disjunct distributions with subtribes Hydromyina (new rank, for the most recent common ancestor [MRCA] of *Anisomys*, *Hyomys*, *Lorentzimys*, *Macruomys*, *Mallomys*, *Pogonomys*, *Pseudomys*, *Uromys*, and the type genus *Hydromys* and all of its descendants) and Rhynchomyina, (new rank, for the MRCA of *Chrotomys*, *Archboldamys*, *Soricomys*, *Apomys* and the type genus *Rhynchomys* and all of its descendants) rather than “the Australasian radiation” or “the shrew-rat clade,” respectively.

The diverse Australian mouse genus *Pseudomys* appeared paraphyletic; however, support values for the relevant nodes were weak and most species were only represented by *cytb*. Further sampling is needed to test these results.

The phylogenetic results reinforce confidence in the placement of the diurnal otomyines (murine lineage 6) as closely related to fellow African Arvicanthini [1, 13, 19, 55, 59, 60]. Basal relationships within the vlei rats *Otomys* (Southern Africa), particularly with respect to the whistling rats *Parotomys* and the Karoo rats *Myotomys*, were poorly supported, again because most species of *Otomys* and *Myotomys* were sequenced only for *cytb*.

*Mus* and the Eurasian field mice *Apodemus* are relatively old genera, with MRCAs dated to 6.4 and 6.0 mya respectively. These compare to the large (16–70 species), but young, genera *Rattus* (2.8 mya), Asian white-bellied rats *Niviventer* (3.5 mya), Asian spiny-rats *Maxomys* (4.8 mya), *Otomys* (2.7–3.7 mya), African soft-furred mice *Praomys*, (3.2 mya), and *Pseudomys* (3.1–3.6 mya).

### Phylogenetics: Cricetidae

Within the hamster family Cricetidae, the earliest split occurred approximately 14.6 mya and led to two clades; the Palearctic Cricetinae (hamsters) plus Holarctic Arvicolinae (voles and lemmings; Fig 3, Section D) on the one hand and the entirely New World clade of Neotominae, Tylomyinae, plus Sigmodontinae (Figs 1 and 3) on the other. The diversification of all five lineages occurred in less than a 1 my interval (Fig 5). Despite those short branches, bootstrap support for these two clades was moderate to high; 96% and 80%, respectively. Monophyly of all five subfamilies was strongly supported. The Cricetinae were robustly resolved throughout, with the notable result that the dwarf hamsters *Cricetulus* are polyphyletic. In particular, *C*. *kamensis* from the Tibetan plateau was more closely related to the Siberian hamster *Phodopus* than to congenerics, and *C*. *migratorius* was closer to the European hamster *Cricetus*. These results echo the concerns of Musser and Carleton [21] who cautioned that the assignment of species in *Cricetulus* to other genera has been in flux and that a comprehensive revision was needed. *Cricetulus kamensis* was placed in *Urocricetus* by Saturnin [61] and Neumann et al. [62] anticipated *Cricetulus* paraphyly given weak support for *C*. *(Urocricetus) lama* being sister to *Phodopus* based on 12S sequences [63].

The earliest split within the Arvicolinae was very recent at 7.0 mya, between the long-clawed mole vole *Prometheomys* and all other genera, with the next radiation poorly resolved among five well-supported clades: (1) the large-bodied muskrats *Neofiber* and *Ondatra*, (2) the lemmings *Lemmus*, *Myopus*, and *Synaptomys* (tribe Lemmini), (3) tree voles *Arborimus* and heather voles *Phenacomys*, that together might form the Dicrostonychini [64] with, (4) collared lemmings *Dicrostonyx*, and (5) all remaining arvciolines, including the Arvicolini, Myodini, Ellobiusini, and Lagurini. The rooting of Arvicolinae here was very different from the well-sampled study of Buzan et al., [65], but that latter study analyzed only mitochondrial *cytb* that has shown to have saturation issues at these divergence dates [13], and resolution may also be complicated by the short internodes in this region. The systematics of this subfamily has been particularly problematic and unstable, with conflict between various morphological studies and mitochondrial DNA [21], as well as between more recent studies that use different combinations of mitochondrial and nuclear genes (e.g., [64, 65, 66, 67]). Key results within clade 5 were (Fig 3 Section D): that red-backed voles *Myodes* were likely paraphyletic with respect to mountain voles *Alticola*, confirming often cited close association [21]; monophyly of the vole genus *Eothonomys* was well-supported; most genera were monophyletic with the exceptions of the possibly polyphyletic voles *Lasiopodomys* (although we note that none of the branches separating the two clades of this genus were well-supported) and the expected paraphyly of the highly speciose voles *Microtus*. The results corroborated the common observation that the base of *Microtus* was an exceptionally rapid and recent diversification (> 62 species in 4.0 million years). Although our increased taxon and gene sampling improved the resolution over prior studies, clearly more data are needed to resolve this region of the tree. Given the long and diverse debates about systematics in the Arvicolinae, further details and taxonomic recommendations will be dealt with elsewhere (Conroy et al., in prep).

Relationships among the three New World subfamilies, Neotominae (Fig 4, Section E), Tylomyinae (Fig 4, Section F), and Sigmodontinae (Fig 4, Sections F and G) remain uncertain, with moderate support (85%) for the grouping of Central and northern South American Tylomyinae with predominantly South American Sigmodontinae to the exclusion of the North American Neotominae. Resolution among these three clades varies with gene analyzed, and many more genes are likely needed for confident resolution.

The basal split at 10.3 mya in Neotominae separates the woodrat tribe Neotomini (*Neotoma*, *Hodomys*, and *Xenomys*; *Nelsonia* was not sampled) from the remaining three tribes. The large majority of diversity fell into the Peromyscini (e.g., deer mice *Peromyscus* and harvest mice *Reithrodontomys*) that was well supported as the sister group to Baiomyini (composed of the pygmy mice *Baiomys* and the singing mice *Scotinomys*). The placement of the monotypic golden mouse *Ochrotomys* as sister to that tribal pair was only moderately supported (BS 78%). Speciose *Neotoma* was monophyletic as was *Reithrodontomys*, but the pivotal genus *Peromyscus* had moderate support for being paraphyletic with respect to the Michoacan deer mouse *Osgoodomys*, giant deer mice *Megadontomys*, Mexican volcano mouse *Neotomodon*, Florida mouse *Podomys*, and deer mice *Habromys*. Numerous papers have tackled the systematics of *Peromyscus* and others have noted its apparent paraphyly, e.g., [68–71], and the debate is too extensive to summarize here. Although most individual nodes affecting the monophyly of the genus had poor to only moderate support, returning *Peromyscus* to monophyly would require many rearrangements, and it seems unlikely that all of these rearrangements would be corroborated by additional data. Taxonomic considerations of our results are discussed in more detail elsewhere (Miller et al., in prep).

Sigmodontinae, the second largest mammalian subfamily, was split basally into two major branches (Fig 4, Sections F and G), with the divergence dated around 10.8 mya. Sigmodontalia consists of the cotton rats *Sigmodon* (the sole genus in Sigmodontini) and the several genera of fish-eating rats of the Ichthyomyini, including here the crab-eating rats *Ichthyomys* and the water-mice *Rheomys*. Both of these tribes contain species distributed in either Central or northern South America. The other major branch was Oryzomyalia and as seen repeatedly before [1, 9, 19, 72–75], the base of which was a very rapid radiation with poor phylogenetic resolution. The root of Oryzomyalia is reconstructed as South American [1] and dated to 8.6 mya, well before the final closure of the Panamanian landbridge after 4 mya [76] or 3 mya [77]. As with the core Murinae, we identified the major lineages of this radiation by labeling 12 tribal-level clades (boxed numbers, Fig 4). These are: (1) the cony rats Reithrodontini, (2) Thomasomyini, a large clade of mostly arboreal species including the Oldfield mice *Thomasomys*, climbing mice *Rhipidomys*, and montane mice *Aepeomys*, (3) the ecologically and geographically diverse rice rats Oryzomyini with over 30 genera, (4) the monotypic Altiplano chinchilla mouse *Chinchillula*, (5) Andean mouse *Andinomys* and Puna mouse *Punomys*, (6) the Andean clade of chinchilla mice *Euneomys*, Chilean climbing mouse *Irenomys*, and Andean swamp rat *Neotomys*, (7) the red-nosed mice *Wiedomys*, (8) *Juliomys*, (9) the Abrotrichini of the southern cone, (10) Atlantic forest rats *Delomys*, (11) the leaf-eared mice *Phyllotis* and their relatives in Phyllotini, and (12) the large and diverse Akodontini that includes field mice *Akodon* and the giant burrowing rats in *Kunsia*. These clades have generally been recovered by other studies using subsets of these genes (primarily *cytb* and *Rbp3*), and all 12 had diverged from each other by 6.7 mya, approximately 1.9 million years after the origin of Oryzomyalia (Fig 5). *Akodon* is the largest genus with over 40 species and dated to 3.9 mya provided it is considered to include *A*. *serrensis* and the grass mouse *Deltamys*, 3.3 mya if it does not.

In summary, across all Muroidea, a total of 81% of the 125 genera for which we have multiple species are monophyletic; 24 genera are not, but many of those (e.g., *Grammomys*, *Microtus*, and *Akodon*) were poorly supported for the nodes that render them non-monophyletic. The genera for which para- or polyphyly is well supported, and thus taxonomic changes are warranted, include: *Gerbilliscus* with respect to *Gerbillurus* [44, 51]; *Gerbillus* with respect to *Dipodillus* [44, 51]; Philippine *Batomys* with respect to *Crateromys*; Southeast Asian *Maxomys* with respect to *Crunomys*; African *Myomyscus* with respect to *Colomys* and *Zelotomys* (because of the position of *Myomyscus verreauxii* [55]); *Praomys*, and *Mastomys*, as noted by Lecompte et al. [55]; *Volemys* polyphyly; *Myodes* with respect to *Alticola*; *Cricetulus* polyphyly; *Peromyscus* with respect to *Habromys*, *Podomys*, *Neotomodon*, and possibly *Megadontomys* and *Osgoodomys*.

### Comparison to other kilo-species phylogenies

The largest supermatrix study of rodents to date is Fabre et al. [10] that included 815 muroids. Results from that study and this are highly concordant topologically with a few minor exceptions. Our results corroborate the muroid portion of the trees in Fabre et al. [10], and their trees provide an important and useful reference for comparative studies of rodents, however, the analyses here provide deeper taxonomic coverage with nuclear genes and in particular multiple nuclear loci for most species. In contrast, Fabre et al. [10] were limited to previously published data that were dominated by rapidly evolving *cytb* (>90% coverage of their included muroids), and for the nuclear loci, mostly *Rbp3* (401 species, 49% coverage), followed by 206 species for *GHR* and 142 species for *RAG1*. Coverage for the latter two genes was more taxonomically clustered. Consequently, the nodal support values in our study were higher, especially at intermediate depths, and many regions have improved from ambiguous to well-supported resolution. The topological concordance between studies appears to be in part a consequence of minimal conflict among genes, allowing even limited genetic sampling to yield trees very close to those from larger datasets. However, dating analyses were less concordant with those here. Fabre et al. [10] used BEAST [28] with a taxonomically partitioned (compartmentalized) approach, with the entire tree subsequently reconstructed from eight hierarchically nested trees, each derived from submatrices. We chose to use the ML tree and apply penalized likelihood as an alternative to compartmentalization, both approaches being solutions to a lack of convergence with Bayesian approaches on the entire data set. Fabre et al. [10] also used only three fossil calibrations, all external to Muroidea, two of which were in slower evolving regions of the rodent tree [78]. All 28 of our calibrations were within the more rapidly evolving Myodonta [1, 9]. The absence of calibrations within Muroidea may have resulted in an underestimation of muroid molecular rates by Fabre et al. [10], resulting in overestimating ages, particularly in the middle and distal regions of the tree, compared to the results found here. In the Fabre et al. [10] chronogram, almost all subfamilies had diverged before the Miocene at 23 mya, and several MRCAs of subfamilies date to the lower Miocene or upper Oligocene, including Sigmodontinae, Murinae, Gerbillinae, and Nesomyinae. In contrast, the results here show most subfamilial divergence occurring between 15 and 20 mya in the lower to middle Miocene, with their respective MRCAs (crown-group roots) dating to between 7 and 13 mya (Fig 1). The MRCA of Eumuroida was placed at approximately 28.5 mya [10], compared to 20.2 mya here. Our Beast results are generally similar to the penalized-likelihood results, with somewhat more recent dates (S7 File).

There are a small number of topological differences to note. Fabre et al.’s chronogram in their Fig 2 grouped Calomyscidae as sister to Nesomyidae, rather than to all other eumuroids as seen in their Supplemental cladogram and the results here. The thomasomyine *Rhagomys* grouped with *Juliomys* (node 8, Fig 4 Section F) rather than with the expected Thomasomyini (node 2, Fig 4), *Delomys* grouped with basal *Reithrodon* (node 1, Fig 4) rather than in its well-supported (BS > 98%) position sister to Phyllotini (node 11, Fig 4). Significant differences include the multiple nodes within the *Cricetulus/Cricetus* clade of hamsters, *Melasmothrix* as sister to the *Rattus/Berylmys* clade instead of the well-supported position sister to *Niviventer/Leopoldamys* clade [54, 55], and *Leopoldamys* sister to *Niviventer/Dacnomys* rather than the well-supported position sister to *Dacnomys*. There are also differences between the two studies in a variety of regions that are poorly supported by either study (e.g., basal Deomyinae Fig 2; among scapteromyines of the Akodontini, node 12, Fig 4).

Our pre-analysis screening of potential sequence data (see Methods) identified several suspect sequences that were included in Fabre et al. [10]—as well as in other studies including [73, 74, 79, 80]—that we excluded from our analysis. These suspect sequences included: the sigmodontine fish-eating rat *Neusticomys monticolus* (GenBank accession EU649036, a chimeric sequence derived from two tribes as reported by Hanson et al., [81]; Steppan and Schenk, unpublished analysis); the sigmodontines *Andalgalomys olrogi* (AY070231) and *Auliscomys boliviensis* (AF387810; both failed to group with congeners or even in Phyllotini and appear to be pseudogenes); and the gerbilline *Meriones shawi* that does not group with congenerics, unlike our *M*. *shawi*. These problematic sequences should be pruned from future analyses that use trees from those studies.

In contrast to the strong concordance among all DNA sequence-based studies, including [10], the two primary supertree studies disagree in many respects with each other and with this study. We compared our results to the widely used supertree from Fritz et al., ([7]; an updated analysis of Bininda-Emonds et al., [6]; hereafter referred to as “Fritz tree”), that along with [6] has been cited more than 1,294 times (ISI accessed October 25 2016), as well as the mammalian portion of the TimeTree of Life [8], hereafter referred to as "TimeTree." The former used matrix representation with parsimony (MRP) to estimate a topology, the latter used more complex algorithms to reconcile both dates and topology from dated ultrametric source trees. Both published trees resolved most genera as monophyletic, and most of those genera were also recovered as monophyletic here. In part, the monophyly of genera in supertrees is a function of assumptions made in their construction, and not because of definitive support found in the underlying source trees. The Fritz tree included 1,396 species of muroids, but resolved only 28.4% of the possible nodes. Large regions were left as polytomies, particularly within genera or among genera within subfamilies. More importantly, only 12.5% of possible nodes agreed with well-supported nodes from this or other multigenic (supermatrix) original analyses (here after considered “corroborated” for simplicity) while 6.9% conflicted with corroborated nodes. Another 7.4% of possible nodes could not be evaluated because they included species not sampled for any published molecular data (many might have been supported by morphological studies). In other words, for the Fritz tree, 72.6% of nodes were unresolved, and of those that were, 45% were supported by multigene molecular phylogenies while 55% were not. We also summarized agreement at deeper levels. The Fritz tree recovered the monophyly of only nine out of 29 multigeneric tribes, 10 of 14 mulitgeneric subfamilies, and none of the four muligeneric families, all clades that were strongly supported here and in many other studies [1, 9, 19, 53, 55, 73].

The TimeTree ([8]; accessible at http://www.biodiversitycenter.org/ttol) was completely resolved, but by necessity from lack of taxon sampling in source trees much of the resolution among what had been polytomies in the Fritz tree do not appear to be based on evidence from source trees. Summary tabulation of agreement with our tree is very similar to the Fritz tree, correctly recovering only nine out of 29 multigeneric tribes, 11 of 14 multigeneric subfamilies, three of four multigeneric families (all but Muridae), and just 42 out of approximately 249 possible supergeneric clades (17%; the denominator calculated as the minimum number of corroborated supergeneric clades present in our tree that can test those in the supertrees). However, these statistics understate the full scope of the conflict. Not only were most tribes incorrectly recovered as non-monophyletic, many of those were highly polyphyletic, especially in the two largest subfamilies, Murinae and Sigmodontinae. Regarding some of the most important and, in our study, well-supported clades, the following tribes were split by the TimeTree into the following number of disparate clades; southeast Asian Rattini, 14 clades; African Arvicanthini, 10 clades (several sister to parts of Rattini); Sahulian/Philippine Hydromyini, five clades; African Praomyini, four clades; Gerbillini, four clades; and among South American sigmodntines, field mice Akodontini, nine clades; Thomasomyinae (that contains only five genera), five clades; Phyllotini, four clades; and Oryzomyini, four clades. In the Arvicolinae: the voles Arvicanthini, five clades; and in the North American Neotominae; Reithrodontomyini, four clades, and Neotomini, three clades. Even the highly divergent and distinctive Philippine cloud runners Phloeomyini (with a 3.6 my long stem lineage, Fig 5) were split into two widely separated clades. The subfamily Dendromurinae was split into three clades. In contrast, both supertrees fully resolved the Malagasy Nesomyinae (seven genera) nearly identical to this study for species in common, and major parts of Neotominae and Arvicolinae are congruent with multigene studies. Other regions of those mammalian supertrees are much more congruent with the best source phylogenies (e.g., Carnivora, Cetartiodactyla), but we caution that users interested in the muroids may find the Fritz and TimeTree phylogenies on balance positively misleading.

Estimated divergence dates in previously published supertrees were also significantly older than those estimated here. Several key examples are highlighted in Table 2. The muroid root is nearly the same for all studies, but differences become pronounced in the middle and distal regions of the tree. The dates estimated here are usually close to the estimates from the fossil record, although it can be difficult to compare directly because some early fossils may be members of stem lineages rather than crown groups (e.g., fossil *Copemys* for extant *Peromyscus*). The most extreme differences include Nesomyidae (42.0 mya in TimeTree versus 15.6 and 11.1 mya in this study and earliest fossils, respectively), and *Rattus*, reconstructed as quite old in the Fritz tree because of polyphyly (29.4 mya) compared to 2.9 and 3.6 mya in this study and the fossil record, respectively. A particularly notable case is the vole subfamily Arvicolinae, that because of their distinctive and ever-growing molars, have a better fossil record than most muroid groups. The supertrees estimate the MRCA at 19.6 and 19.8 mya for the Fritz tree and TimeTree, respectively, in contrast to 7.0 and 4.9 mya for this study and the earliest fossils, respectively.

**Table 2:** Divergence dates for the most recent common ancestors of representative clades.

Clade	Fritz et al., (2009)	Hedges and Kumar (2015)	Schenk et al. (2013)	This study	Maximum First Appearance (PBDB)
Myodonta	70.3	59.1	46.4	48.6	48.6
Muroidea	48.0	47.8	43.3	45.2	37.2
Eumuroida	na	43.2	25.1	20.0	28.4
Spalacidae	na	41.5	26.5	23.6	16.0
Rhizomyinae	14.3	15.3	8.3	8.0	9.8^a^
Nesomyidae	na	42.0	17.6	15.6	11.1
Cricetidae	na	40.2	17.7	14.6	37.2^b^
Muridae	na	35.7	20.7	17.4	28.4^c^[82]
Gerbillinae	23.7	23.6	10.2	10.2	23.0 ^d^[83, 84]
Sahulian Hydromyini	na	na	6.1	6.0	no fossils
*Mus*	13.7	10.2	5.9^e^	6.4	5.3
*Rattus* sensu lato	29.4^f^	14.4^f^	2.4	2.8	3.6
Arvicolinae	19.6	19.8	9.2	7.0	4.9
*Microtus*	9.0^f^	9.8^f^	3.4	4.0	2.6
*Peromyscus* sensu lato	20.0	12.5	5.1	5.4	13.6^g^
Sigmodontinae	22.8	27.5	11.6	10.8	5.3
Oryzomyalia	16.3	27.5^f^	7.8	8.6	4.0

Dates estimated for the supertrees in Fritz et al., [7] and Hedges and Kumar [8], compared to Schenk et al. [1] and this study. Maximum First Appearance is the earliest fossil member of the crown group, calculated by Paleobiology Database [31], except where otherwise noted. na = Not applicable because taxon was not monophyletic in the cited study.

^a^Lopez-Antonanzas & Flynn [45].

^b^Likely includes muroid stem lineages (fossil attributed to Cricetidae was the same as for earliest Muroidea).

^c^PBDB estimate based on the age of *Tachyoryctoides* being a murid, but Flynn [82] considered this genus to be enigmatic and a primitive muroid, and thus not likely outside crown Muridae.

^d^PBDB age based on a fossil identified as *“Gerbillidae indet*.*”* by Thomas et al. [83] dated to the lower Miocene (16–23 mya). This fossil could be a stem lineage. The PL estimate for the divergence of Gerbillinae from its sister group at 16 mya agreed with the paleontological assessment of Tong and Jaeger [84] of 16 mya.

^e^Not all basal lineages were sampled in cited study, date represents MRCA of a subclade.

^f^Taxon was polyphyletic in cited study, date for MRCA including other taxa.

^g^Earliest appearance is for a member of *Copemys*, an extinct genus that is generally considered ancestral and paraphyletic to *Peromyscus* and therefore the PBDB date does not correspond to the crown group.

### Diversity over time

**Fig 6:**
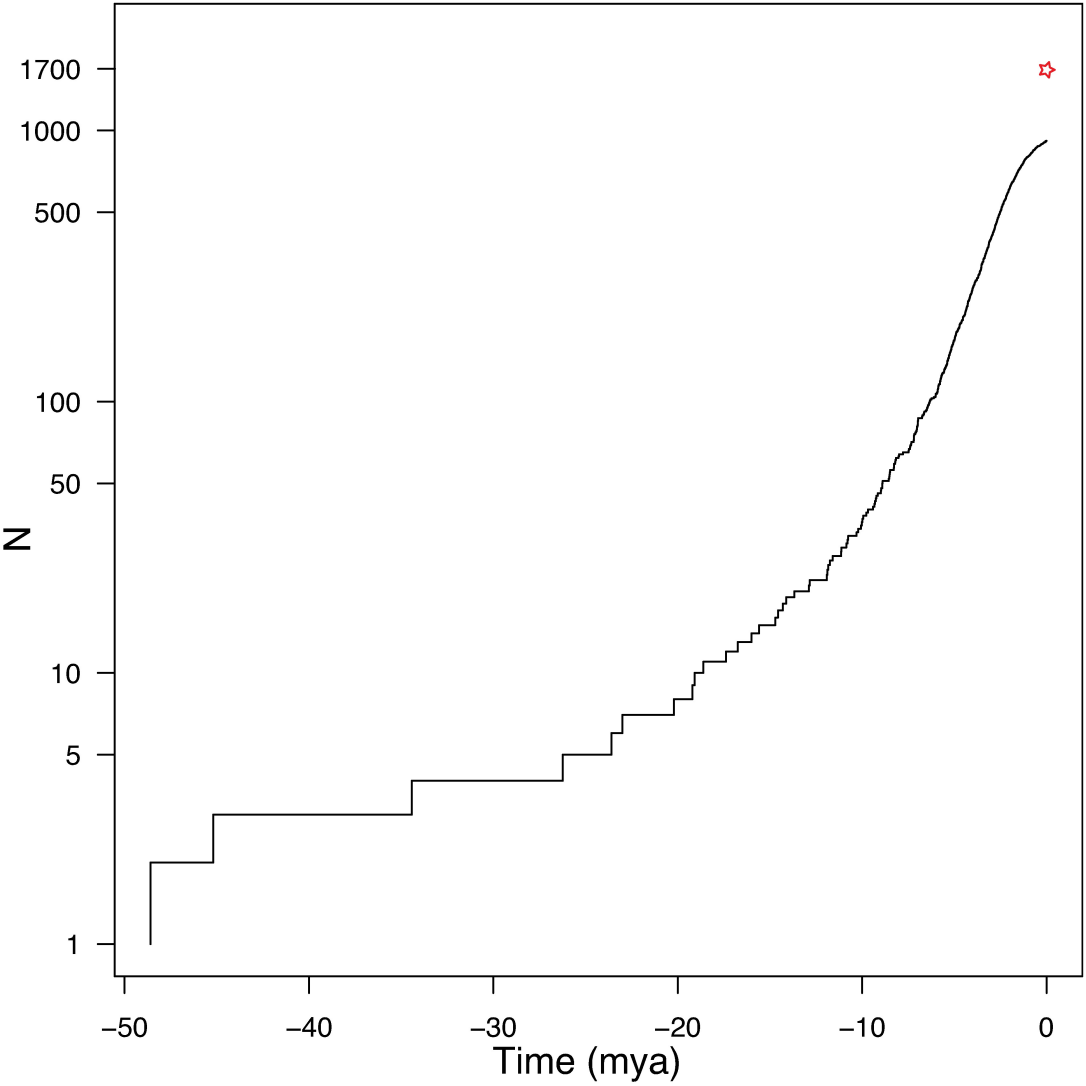
Lineage-through-time (LTT) plot of muroid diversity demonstrating an accelerating diversification rate. Red star indicates an estimate of total muroid diversity (1,700 species), assuming future discoveries of species will increase the total beyond the current count of approximately 1,620.

**Fig 7:**
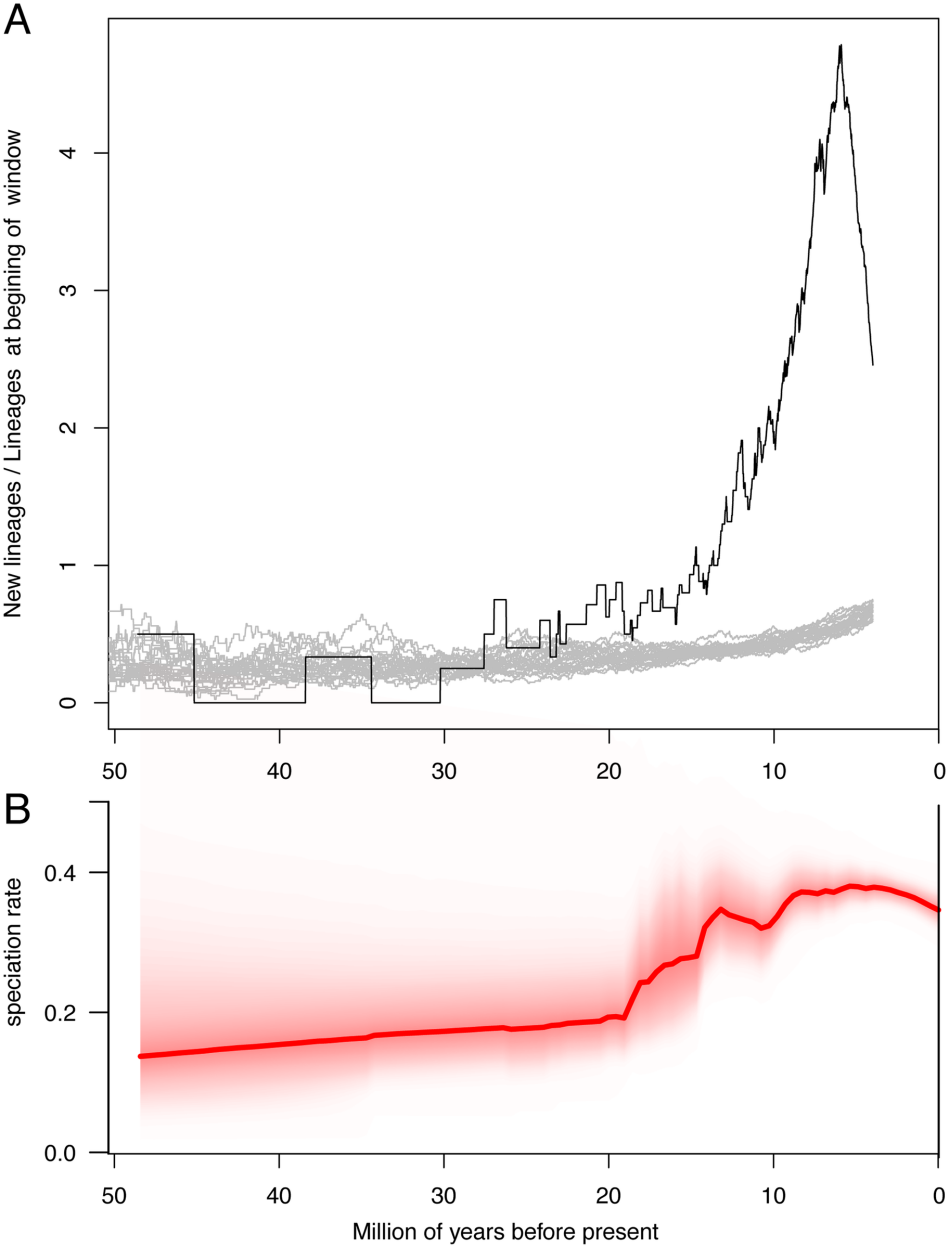
Sliding-window and BAMM analyses of muroid diversification demonstrating an accelerating diversification rate. (A) Sliding-window analysis: vertical axis corresponds to the average among-lineage net diversification rate. A value of 1.0 corresponds to a doubling of diversity per 2-million years. Grey lines illustrate 100 simulations under an extinction rate of 0.1/million years. (B) BAMM analysis: solid red line indicates median estimate, fine red lines (cloud) indicate probability distribution.

The lineage-through-time (LTT) plot indicated an accelerating net diversification rate through time with no obvious inflection point (Fig 6). Constant diversification rate would yield a straight line on a semi-log plot. The tailing-off of the curve in the last 3 million years is likely a function of incomplete sampling of species within genera. This cumulative pattern contrasts with that seen within subclades of muroids, where none of the clades arising after continental colonizations deviated significantly from constant rates, with the exception of the South American Oryzomyalia (Sigmodontinae) and Sahulian Hydromyini (Murinae) that declined over time [1]. The sliding-window analysis (Fig 7A), that effectively estimates the slope of the LLT though time in 2-million-year windows, clearly shows the magnitude of the diversification rate increase and revealed more subtle patterns. There were several weak bursts of diversification, seen around 23–19 mya, 12 mya, and 7 mya. The Bayesian estimated rates show a very similar pattern, with an increase at 19 mya and then peaks at 13 mya and 5–9 mya (Fig 7B). These corresponded with the overlapping basal diversifications of Spalacidae and Eumuroida (the rate increase estimated by BAMM only corresponds to Eumuroida, Fig 7B), then the clustering of basal radiations of several subfamilies (e.g., Murinae, Nesomyinae, and Neotominae), and the Oryzomyalia radiation, respectively. However, the rise in rates was not caused just by the greatest diversifications (Murinae and Oryzomyalia), because the increase, though smaller, remains even if those clades are pruned from the tree (unpubl. data). The rapid decline in rate over the last 5 million years (Fig 7A, less rapid in Fig 7B) is a function of incomplete sampling of species within genera. The large peak in rates towards the recent was significantly greater than expected under constant rates with moderate extinction and speciation rates (grey lines, Fig 7A). Increasing the extinction rate in simulations results in a larger surge (upturn) in net diversification towards the recent, and the extinction rate needed to approximate the curve seen in our tree is quite high, around 0.2–0.3/mya.

**Fig 8:**
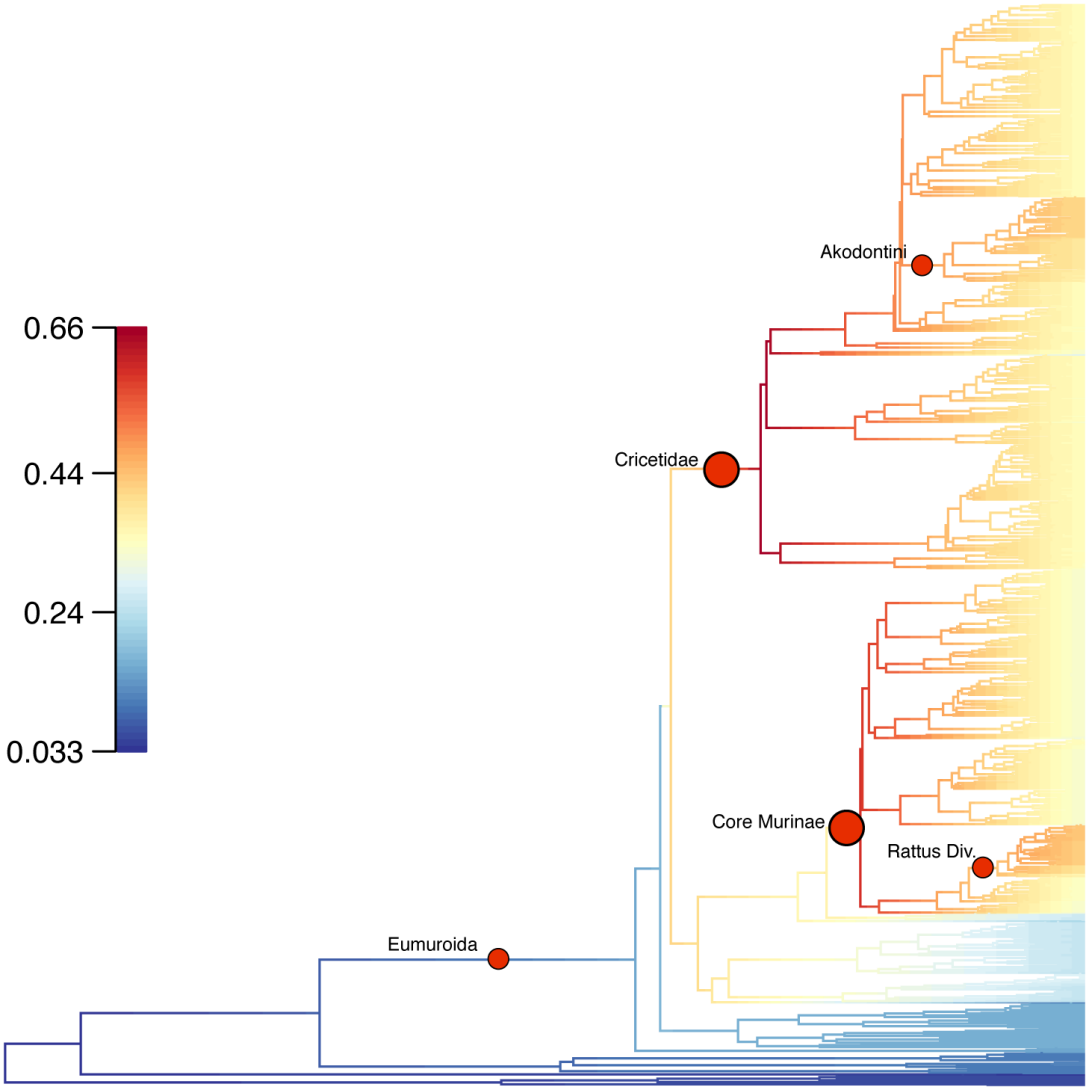
Muroid chronogram with branches colored by net diversification rate estimated by BAMM. Large red dots indicate positions of the two rate shifts found in all regimes within the 95% credibility sets and the small red dots are those shifts found in some of the regimes within the 95% credibility sets. These correspond to the following clades (from oldest to youngest): Eumuroida, Cricetidae, core Murinae (sensu Steppan et al., [9]), Akodontini, and Rattus Division.

Bayesian estimates of diversification rate shifts consistently identified two nodes in the 95% credibility set; Cricetidae and core Murinae (Fig 8). Core Murinae was also identified by a variety of additional rate shifts methods in a 300 species analysis but interestingly Cricetidae was not [1]. Three other nodes appeared in some of the regimes contained in the 95% credibility set, and these correspond to Eumuroida, Akodontini, and the Rattus Division of Murinae (Fig 8). Schenk et al. [1] also identified Eumuroida but not the latter two, and notably, the strongest supported rate increase in that study, Oryzomyalia, was not identified by BAMM. Significantly increasing rates were not seen in any of the 28 individual continental radiations analyzed by Schenk et al. [1]. These two sets of results may be reconciled if across muroids there has been an increase in the frequency of intercontinental dispersal—perhaps due to changing global environmental conditions that favor small rodents—or higher extinction rates than typically estimated by diversification analyses. High extinction rates, especially early in muroid history, would have pruned off most of the early lineages, leaving disproportionately long branches compared to the last several million years, during which time extinction would not yet have had the opportunity to eliminate as much of the diversity. Alternatively, the estimated deviation from constant diversification could be due to distortion of the relative branching events in the estimated chronogram. Shifting the middle nodes of the tree towards the root would diminish the apparent increase in overall rates, but would only eliminate it when stretched to the point that the chronogram would obviously deviate from relative branch lengths in the original ML phylogram. For example, increasing rates are recovered from chronograms estimated for all smoothing-functions with penalized likelihood except for the most extreme (10,000) that was rejected by r8s as an unreliable reconstruction (unpbl. results). A counter argument to increasing rates simply being an artifact of analyzing a large tree that extends deep into time is that other large clades do not generally show such a pattern. Nearly constant rates are typically seen in groups as varied as all eukaryotes and major subgroups [45] to funariid birds [8], and in other cases rates are estimated to decline as in bats [85] and many groups in general, e.g., [86, 87]. We propose that the most likely explanation is a combination of some increase in net diversification rate coupled with a high average extinction rate. The ultimate resolution of this question would require a much more complete paleontological record of muroid diversity than we have at the present.

### Conclusions

Muroidea is the most spectacular family-level radiation in mammals and a scientifically important group, frequently used in biomedical studies. These biomedical studies are becoming more frequently comparative in nature, and an accurate and robust phylogeny is critical for further advancement. Here we have estimated the most extensive hypothesis to data on the clade, in terms of combined taxon and character sampling. Rigorous analyses have clearly and robustly delineated the genealogical framework, providing a hypothesis for comparative studies and the basis for taxonomic revisions. The history of muroids has been marked by several distinct bursts of diversification (although not all methods of analysis are fully concordant). An overarching pattern of increasing diversification rate spans across multiple clades and calls for more detailed tests, particularly to quantify to what extent a high background extinction rate may play in producing that pattern. However, diversification analyses here and elsewhere [1, 11] still have not provided a clear explanation for the evolutionary success of this clade compared to other mammals.

## Supporting information

S1 Appendix GenBank accession and museum voucher numbers for samples used in phylogenetic analyses. (PDF)

S1 Fig Maximum likelihood phylogram for *Acp5*. (PDF)

S2 Fig Maximum likelihood phylogram for *BRCA1*. (PDF)

S3 Fig Maximum likelihood phylogram for *GHR*. (PDF)

S4 Fig Maximum likelihood phylogram for *Rbp3*. (PDF)

S5 Fig Maximum likelihood phylogram for *RAG1*. (PDF)

S6 Fig Maximum likelihood phylogram for *cytb*. (PDF)

S7 Fig BEAST chronogram from concatenated data. (PDF)

S1 File Maximum likelihood phylogram for *Acp5* in Newick format. Scale bar equals probability of substitution.
(TRE)

S2 File Maximum likelihood phylogram for *BRCA1* in Newick format. Scale bar equals probability of substitution.
(TRE)

S3 File Maximum likelihood phylogram for *GHR* in Newick format. Scale bar equals probability of substitution.
(TRE)

S4 File Maximum likelihood phylogram for *Rbp3* in Newick format. Scale bar equals probability of substitution.
(TRE)

S5 File Maximum likelihood phylogram for *RAG1* in Newick format. Scale bar equals probability of substitution.
(TRE)

S6 File Maximum likelihood phylogram for *cytb* in Newick format. Scale bar equals probability of substitution.
(TRE)

S7 File BEAST chronogram from concatenated data in Newick format. (TRE)
