# Supplementary material for: Muroid rodent phylogenetics: 900-species tree reveals increasing diversification rates
Source: PLoS One. 2017 Aug 16;12(8):e0183070. doi: 10.1371/journal.pone.0183070 (PMC5559066; doi:10.1371/journal.pone.0183070)
Supplement: S1 Appendix — (PDF) [file pone.0183070.s001.pdf]

S1 Appendix. GenBank accession and museum voucher numbers for samples used in phylogenetic analyses.

Taxon			Gene			
	Acp5	*BRCA1*	*GHR*	*RBP3*	*RAG1*	*cytb*
Abeomelomys sevia	EU349607	EU349682	EU349793	EU349832	EU349879	KY753930<br>KU 161033
Abrothrix andinus	—	KC953150	KC953231	KC953345	KC953467	KY753931<br>MVZ 174066
Abrothrix hershkovitzi	—	—	—	—	—	EU840992
Abrothrix jelskii	—	KC953151	KC953232	KC953346	KC953468	KY753932<br>VPT 1886
Abrothrix lanosus	—	—	—	EU683430	—	—
Abrothrix longipilis	MF110300<br>MVZ 154494	KC953152	KC953233	—	KC953469	EU579470
Abrothrix olivaceus	MF110301<br>MVZ 182589	—	—	MF097704<br>MVZ 182589	—	AF297879
Abrothrix xanthorinus	—	—	KC953234	—	KC953470	—
Acomys cahirinus	—	—	FN984742	FN984743	—	FJ415482
Acomys chudeaui	—	—	—	—	—	FJ415488
Acomys cilicicus	—	—	—	—	—	FJ415481
Acomys dimidiatus	—	—	—	—	—	Z96062
Acomys ignitus	—	AY295008	AY294923	KC953348	AY294951	JN247674
Acomys johannis	—	—	—	—	—	AJ010567
Acomys minous GroupA	—	—	—	—	—	AJ233951
Acomys minous GroupB	—	—	—	—	—	GU046553
Acomys nesiotes	—	—	—	—	—	AJ233952
Acomys perisvalli	—	—	—	—	—	KR089012
Acomys russatus	—	—	FM162071	FM162053	—	Z96066
Acomys spinosissimus	—	—	—	AY326074	—	KR089013
Acomys subspinosus	—	—	—	—	—	AJ010557
Acomys wilsoni	—	—	—	—	—	AJ010561
Aegialomys galapagoensis	—	—	—	EU649039	—	EU579478
Aegialomys xanthaeolus Ecuador	—	—	—	—	—	EU340015
Aegialomys xanthaeolus Peru	—	—	KC953234	KC953349	KC953470	KY753933<br>MVZ 145540
Aepeomys lugens	—	—	—	DQ003722	—	—
Aethomys chrysophilus	—	—	—	AY326075	—	AJ604515
Aethomys kaiseri	—	—	—	—	—	AJ604514

Akodon aerosus	MF110302<br>MVZ 172847	—	KC953235	KC953350	KC953471	AKOMTCYTBQ
Akodon affinis	—	—	—	—	—	AY196164
Akodon albiventer	—	—	—	—	—	AY494838
Akodon azarae	MF110303<br>TK 49098	—	—	MF097705<br>TK 49098	MF097816<br>TK 49098	DQ444328
Akodon boliviensis	MF110304<br>FMNH 162754	—	KC953236	KC953351	AY294960	KY753934<br>FMNH 162747
Akodon budini	—	—	—	—	—	AY605060
Akodon cursor	MF110305<br>MVZ 182072	—	MF074854<br>MVZ 182072	EF626797	—	EF206814
Akodon dayi	MF110306<br>USNM 584504	—	MF074855<br>USNM 584504	MF097706<br>USNM 584504	MF097817<br>USNM 584504	AY605059
Akodon dolores	MF110307<br>OMNHN 36058	—	MF074856<br>OMNHN 36058	MF097707<br>OMNHN 36058	MF097818<br>OMNHN 36058	AY273904<br>OMNHN 36058
Akodon fumeus	—	—	MF074857<br>TK 14547	MF097708 TK<br>14547	MF097819<br>TK 14547	AY605061
Akodon glaucinus	—	—	—	—	—	EU260483
Akodon iniscatus	MF110308<br>OMNHN 23611	—	MF074858<br>OMNHN 23611	MF097709<br>OMNHN 23611	MF097820<br>OMNHN 23611	AY273917
Akodon juninensis	MF110309<br>MVZ 173038	—	—	—	MF097821<br>MVZ 173038	AKOMTCYTBH
Akodon kofordi	MF110310<br>MVZ 171670	—	KC953237	KC953352	KC953472	KY753935<br>MVZ 171670
Akodon lindberghi	—	—	—	—	—	AF184057
Akodon lutescens	MF110311<br>MVZ 173248	—	KC953238	KC953353	KC953473	KY753936<br>MVZ 173248
Akodon mimus	MF110312<br>MVZ 171752	KC953153	KC953239	AY277425	KC953474	M35710
Akodon molinae	—	—	—	—	—	AY494839
Akodon mollis	MF110313<br>TK 104487	—	MF074859<br>TK 104487	MF097710<br>TK 104487	MF097822<br>TK 104487	U03546
Akodon montensis	MF110314<br>MVZ 197459	—	—	AY277426	MF097823<br>MVZ 197459	AF184055
Akodon mystax	—	—	—	—	—	AY273907
Akodon orophilus Amazonas	—	—	—	—	—	U03524
Akodon orophilus Junin	—	—	—	—	—	KY753937<br>MVZ 173045
Akodon paranaensis	—	—	—	EU649035	—	EF622506
Akodon philipmyersi	—	—	—	—	—	AY702965

Akodon reigi	—	—	—	—	—	AY195865
Akodon serrensis	—	—	—	AY277429	—	AY273908
Akodon siberiae	—	—	—	AY277430	—	AY273909
Akodon simulator	MF110315<br>OMNHN 30014	—	—	—	—	EU260481
Akodon spegazzinii	MF110316<br>OMNHN 36501	—	—	MF097711<br>OMNHN 36501	MF097824<br>OMNHN 36501	KY753938<br>OMNHN 36501
Akodon subfuscus	—	—	—	—	MF097825<br>LAC 89	M35695
Akodon sylvanus	—	—	—	—	—	DQ683180
Akodon tartareus	—	—	—	—	—	EU260485
Akodon toba	MF110317<br>OMNHN 37447	—	—	AY277433	MF097826<br>OMNHN 37447	U03527
Akodon torques	MF110318<br>MVZ 171720	KC953154	KC953240	KC953354	KC953475	M35700
Akodon varius	—	—	—	—	—	EU260478
Allactaga elater	—	—	—	JF835103	—	AJ389534
Allactaga sibirica	MF110319<br>USNM 449152	AY294996	AY294897	AY326076	AY241467	KY753939<br>USNM 449152
Allocricetulus eversmanni	—	—	—	—	—	AJ973378
Alticola argentatus	—	—	—	—	—	DQ845186
Alticola barakshin	—	—	—	—	—	DQ845188
Alticola lemminus	—	—	—	—	—	AF7474
Alticola macrotis	—	—	—	—	—	ZINRAS_90980
Alticola semicanus	—	—	—	—	—	DQ845192
Alticola strelzowi	—	—	—	—	—	DQ845190
Amphinectomys savamis	—	—	—	AY163579	—	EU579480
Andalgalomys pearsoni	MF110320<br>MSB 55245	KC953155	KC953241	KC953355	AY963176	AF159285
Andalgalomys roigi	MF110321<br>OMNHN 30015	—	MF074860<br>OMNHN 30015	MF097712<br>OMNHN 30015	MF097827<br>OMNHN 30015	KY753940<br>OMNHN 30015
Andinomys edax	—	KC953156	KC953242	KC953356	MF097828<br>FMNH 162759	AF159284
Anisomys imitator	DQ023440	—	DQ019052	EU349833	MF097829<br>SAMA 45107	KY753941<br>SAMA 45107
Apodemus agrarius	DQ023441	EU349658	DQ019054	AB096842	DQ023472	KY753942<br>MVZ 159220
Apodemus alpicola	—	—	—	AB032861	—	AF159391
Apodemus argenteus	—	—	—	AB032855	—	AB164571
Apodemus chevrieri	—	—	—	AB096847	—	AB096821

Apodemus draco	MF110322<br>USNM 574338	—	—	AB109398	—	JF503106
Apodemus flavicollis	MF110323<br>MVZ 181468	—	AM910943	AB032860	—	JF819967
Apodemus gurkha	—	—	—	AB032859	—	AB032852
Apodemus hermonensis	—	—	—	AB303231	—	—
Apodemus latronum	—	—	GU908445	AB096852	—	AM945834
Apodemus mystacinus	MF110324<br>SP 7861	KC953157	DQ019053	MF097713<br>SP 7861	KC953476	KY753943<br>SP 7861
Apodemus pallipes	—	—	—	AB096854	—	AF160603
Apodemus peninsulae	—	—	—	AB032857	—	AB073809
Apodemus semotus	DQ023442	—	DQ019055	AB032862	DQ023473	KY753944<br>MVZ 180489
Apodemus speciosus	—	—	AB491493	AB032856	—	AB164486
Apodemus uralensis	—	—	—	AB096853	—	FN430750
Apodemus witherbyi	—	—	—	AB032863	KC953477	KY753945<br>MVZ 198798
Apomys abrae	HM371062	—	—	—	KM099834	HM371059
Apomys aurorae	HM371036	—	—	—	KM099836	HM371036
Apomys banahao	HM371079	—	—	—	KM099837	HM371079
Apomys brownorum	HM371087	—	—	—	KM099839	HM371087
Apomys camiguinensis	AY324476	—	—	—	KM099841	AY324477
Apomys datae	AY324463	KC953158	KC878169	EU349836	KC953478	AY324463
Apomys gracilirostris	AY324465	—	—	—	KM099852	AY324465
Apomys hylocoetes	MF110325<br>FMNH 148149	AY295000	AY294915	KC953357	MF097830<br>FMNH 147871	AY324469
Apomys insignis	KM099816	—	—	DQ191492	KM099854	AY324472
Apomys littoralis	—	—	—	—	MF097831<br>FMNH 206109	—
Apomys magnus	HM371043	—	KU375161	MF097714<br>FMNH 183570	KM099856	HM371043
Apomys microdon	MF110326<br>FMNH 167241	—	—	DQ191493	MF097832<br>FMNH 167241	AY324481
Apomys minganensis	HM371046	—	—	—	KM099858	HM371046
Apomys musculus	KM099730	—	—	DQ191494	KM099860	AY324482
Apomys sacobianus	KM099817	—	—	—	KM099861	KM099818
Apomys sierrae	HM371011	—	—	—	MF097833<br>FMNH 191232	HM371011
Apomys zambalensis	HM371016	—	—	—	—	HM371012
Arborimus albipes	MF110327	—	—	—	—	KY753946

	UWBM 79504					UWBM 79504
Arborimus longicaudus	—	—	MF074861<br>UWBM 78048	MF097715<br>UWBM 78048	—	KY753947<br>UWBM 78048
Archboldomys luzonensis	AY687857	EU349675	EU349794	EU349837	DQ023466	EU349736
Archboldomys maximus	—	—	—	JQ898078	—	JQ898033
Arvicanthis abyssinicus	—	—	—	—	—	AF004566
Arvicanthis nairobae	—	—	—	—	MF097834<br>CN 102433	KY753948<br>CN 102433
Arvicanthis neumanni	DQ023425	EU349648	AY294918	KC953358	AY294946	KY753949<br>H 894
Arvicanthis niloticus	—	—	KC953243	DQ022386	—	AF004570
Arvicola amphibius	—	—	AM392380	—	—	AF119269
Auliscomys pictus	MF110328<br>VPT 1882	—	—	—	—	U03545
Auliscomys sublimis	MF110329<br>FMNH 162764	KC953159	KC953244	KC953359	AY294965	U86816
Baiomys musculus	—	—	KC953245	KC953360	KC953479	AF548481
Baiomys taylori	MF110330<br>MSB 46296	—	EF989740	AY277408	—	AF548477
Bandicota bengalensis	—	—	AM910945	AM408331	—	AM408336
Bandicota indica	—	—	—	HM217713	—	KY753950<br>SAMA 64912
Bandicota savilei	—	—	—	HM217665	—	HM217385
Batomys granti	DQ023450	AY295002	AY294917	EU349838	AY241461	AY324459
Batomys salomonseni	—	—	—	DQ191497	—	DQ191471
Beamys hindei	—	AY294998	AY294904	AY326077	MF097835<br>FMNH 150099	KY753951<br>FMNH 166652
Beamys major	—	—	MF074862<br>FMNH 196215	—	MF097836<br>FMNH 196215	KY753952<br>FMNH 196215
Berylmys berdmorei	—	—	—	HM217639	—	HM217432
Berylmys bowersi	MF110331<br>MVZ 186482	KC953160	DQ019056	KC878201	DQ023457	KC878024
Bibimys chacoensis	—	—	—	AY277435	—	—
Bibimys labiosus	—	—	—	AY277436	—	DQ444329
Blanfordimys bucharensis	—	—	AM392392	—	—	AM392369
Blarinomys breviceps Bahia	—	—	—	—	—	AF108668
Blarinomys breviceps D'Elia	—	—	—	AY277437	—	AY275112
Brachiones przewalskii	—	—	—	—	—	AB381903
Brachytarsomys albicauda	MF110332<br>USNM 449351	—	MF074863<br>USNM 449351	AY326078	KC953480	KY753953<br>USNM 449351

Brachytarsomys villosa	—	—	MF074864<br>AMNH 275216	MF097716<br>AMNH 275216	MF097837<br>AMNH 275216	KY753954<br>AMNH 275216
Brachyuromys betsileoensis	MF110333<br>USNM 449216	KC953161	KC953246	AY326079	KC953481	KY753955<br>USNM 449216
Brachyuromys ramirohitra	—	—	GQ272594	—	—	AF160514
Brucepattersonius igniventris	MF110334<br>MVZ 183250	KC953162	KC953247	AY277438	KC953482	KY753956<br>MVZ 183250
Brucepattersonius iheringi	—	—	—	—	—	AF108667
Brucepattersonius soricinus	—	—	—	AY277439	—	AY277486
Bullimus bagobus	—	—	GQ405369	DQ191498	—	DQ191472
Bullimus gamay	—	—	GQ405370	DQ191499	—	DQ191473
Bullimus luzonicus	—	—	GQ405371	DQ191500	—	DQ191474
Bunomys chrysocomus	EU349608	EU349667	EU349795	EU349839	EU349880	KC878025
Bunomys penitus	—	—	MF074865<br>MSB 93130	—	MF097838<br>MSB 93130	KY753957<br>MSB 93130
Calomys callosus	MF110335<br>MSB 13009	KC953163	KC953248	AY277440	KC953483	AF159293
Calomys expulsus	—	—	—	—	—	DQ447290
Calomys fecundus	—	—	—	—	—	AY033166
Calomys hummelincki	—	—	—	—	—	AF385598
Calomys laucha	MF110336<br>UWBM 72276	—	—	—	—	AY964052
Calomys lepidus	MF110337<br>FMNH 162785	KC953164	AY294931	KC953361	AY294966	AF385606
Calomys musculinus	MF110338<br>UWBM 72233	—	—	—	—	AF385604
Calomys sorellus	MF110339<br>MVZ 171542	—	—	—	MF097839<br>MVZ 171542	KY753958<br>MVZ 171542
Calomys tener	—	—	—	—	—	AF385595
Calomys tocantinsi	—	—	—	—	—	DQ447278
Calomys venustus	—	—	KC953249	KC953362	KC953484	KY753959<br>PY 13
Calomyscus bailwardi	—	—	—	—	—	AF160604
Calomyscus baluchi	—	—	GQ405372	AY163581	—	EU135586
Calomyscus hotsoni	—	—	—	—	—	EU135579
Calomyscus sp.	MF110340<br>MVZ 191923	KC953165	AY294901	—	KC953485	KY753960<br>MVZ 191923
Cannomys badius	MF110341<br>SAMA 69096	KC953166	KC953250	KC953363	MF097840<br>SAMA 69096	KY753961<br>SAMA 69096
Carpomys phaeurus	—	—	GQ405373	DQ191501	—	DQ191475

Cerradomys maracajuensis	MF110342<br>USNM 584575	—	—	—	—	AF181278
Cerradomys marinhus	—	—	—	—	—	AF181279
Cerradomys scotti	MF110343<br>USNM 584584	—	—	EU649040	—	EU579482
Cerradomys subflavus	MF110344<br>MVZ 197868	—	KC953251	MF097717<br>MVZ 197868	KC953486	AF181274
Chelemys macronyx	MF110345<br>MVZ 155800	—	KC953252	AY277441	—	U03533
Chilomys instans	—	—	—	—	—	AF108679
Chinchillula sahamae	MF110346<br>VPT 1825	—	—	KC953364	KC953487	—
Chionomys nivalis	—	—	AM392378	AM919424	—	AY513845
Chiromyscus chiropus	EU349609	EU349665	EU349796	EU349840	EU349881	KY753962<br>SAMA 69097
Chiropodomys gliroides	MF110347<br>AMCC 101587	EU349674	EU349797	EU349841	EU349882	KY753963<br>AMCC 101511
Chiruromys vates	MF110348<br>KU 160658	—	—	KC953365	EU349883	EU349741
Chrotomys gonzalesi	AY324461	—	AY294943	EU349843	EU349884	EU349742
Chrotomys mindorensis	—	—	—	JQ898073	—	—
Chrotomys sibuyanensis	—	—	GQ405376	DQ191504	—	AY687862
Chrotomys silaceus	—	—	GQ405377	DQ191502	—	AY687859
Chrotomys whiteheadi	—	—	—	JQ898074	—	AY687864
Colomys goslingi	MF110349<br>CN 102451	—	MF074866<br>CN 102451	MF097718<br>CN 102451	MF097841<br>CN 102451	KY753964<br>CN 102451
Conilurus penicillatus	—	EU349694	DQ019057	EU349844	DQ023467	SAMA 7411
Crateromys heaneyi	—	—	GQ405378	DQ191505	—	DQ191476
Cremnomys cutchicus	—	—	—	DQ022384	—	DQ022381
Cricetomys emini	MF110350<br>FMNH 160394	—	—	AY326081	—	KY753965<br>FMNH 160394
Cricetomys gambianus	MF110351<br>FMNH 166654	KC953167	AY294905	KC953366	AY294936	KY753966<br>FMNH 166654
Cricetulus barabensis	—	—	—	—	—	AJ973384
Cricetulus griseus	—	—	—	AB033705	AY011885	AB033693
Cricetulus kamensis	MF110352<br>USNM 449165	—	MF074867<br>USNM 449165	MF097719<br>USNM 449165	MF097842<br>USNM 449165	KY753967<br>USNM 449165
Cricetulus longicaudatus	—	—	—	AY326082	—	AJ973386
Cricetulus migratorius	MF110353<br>MVZ 191941	—	AY294926	KC953367	AY294956	KY753968<br>MVZ 191941

Cricetus cricetus	KR088976	KC953168	KC953253	AY277410	KC953488	KR089014
Crunomys melanius	—	—	GQ405379	DQ191506	—	DQ191477
Crunomys suncoides	—	—	—	DQ191507	—	DQ191478
Dacnomys millardi	MF110354<br>MVZ 186519	KC953169	DQ019058	KC878206	MF097843<br>MVZ 186519	KC878029
Dasymys incomtus	MF110355<br>SAMA 65735	EU349653	EU349798	KC878207	DQ023459	AF141217
Dasymys rufulus	—	—	—	DQ022387	—	AF141216
Delanymys brooksi	MF110356<br>FMNH 157490	KC953170	KC953254	KC953368	KC953490	KY753969<br>FMNH 157490
Delomys collinus	—	—	—	—	—	AF181282
Delomys dorsalis	MF110357<br>MVZ 182789	—	KC953255	KC953369	KC953491	AF108686
Delomys sublineatus	—	—	—	AY277443	—	AF108687
Deltamys kempi	—	—	—	AY277444	—	AY195860
Dendromus insignis	MF110358<br>FMNH 144355	—	KC953256	KC953370	KC953492	KY753970<br>FMNH 190313
Dendromus mesomelas	MF110359<br>FMNH 161269	AY294997	AY294902	KC953371	AY241458	KY753971<br>FMNH153931
Dendromus mystacalis	—	—	MF074868<br>SAMA 65832	—	—	KY753972<br>SAMA 65832
Dendromus nyasae	—	—	KC953257	KC953372	KC953493	KY753973<br>FMNH 138122
Dendromus nyikae	—	—	—	AY326083	—	—
Deomys ferrugineus	—	AY295007	AY294922	KC953373	AY241460	EU349745
Dephomys defua	—	—	—	MF097720<br>CN 100487	—	KY753974<br>CN 100487
Desmodilliscus braueri	—	—	—	FN357289	—	AJ851273
Desmodillus auricularis	KR088977	KC953171	DQ019048	KC953374	KC953494	KR089015
Desmomys harringtoni	—	—	—	EU292144	—	AF141206
Dicrostonyx groenlandicus	—	—	—	—	—	AF119268
Dicrostonyx hudsonius	MF110360<br>CN 96670	—	MF074869<br>CN 96670	MF097721<br>CN 96670	MF097844<br>CN 96670	KY753975<br>CN 96670
Dicrostonyx torquatus	—	—	AM392381	—	—	—
Dinaromys bogdanovi	—	—	—	—	—	EU190891
Diplothrix legata	EU349612	EU349670	EU349799	AB033706	EU349885	KY753976<br>HS 1163
Dipodillus campestris	KR088978	—	KR089038	—	KR089045	KR089016

Dipodillus dasyurus	KR088979	—	KR089039	MF097722<br>TK 25570	KR089046	KR089017
Dipodillus simoni	KR088980	—	KR089040	—	—	GU356579
Dipus sagitta	—	—	AM407908	AJ427232	—	AM407909
Drymoreomys albimaculatus	—	—	—	EU649042	—	EU579487
Eligmodontia bolsonensis	—	—	—	—	—	EU377644
Eligmodontia hirtipes	—	—	—	—	—	EU377632
Eligmodontia moreni	MF110361<br>OMNHN 34610	—	MF074873<br>OMNHN 34610	MF097723<br>OMNHN 34610	MF097845<br>OMNHN 34610	
Eligmodontia morgani	MF110362<br>MVZ 182670	—	—	—	—	AF108691
Eligmodontia puerulus	MF110363<br>OMNHN 34746	—	MF074874<br>OMNHN 34746	MF097724<br>OMNHN 34746	MF097846<br>OMNHN 34746	EU377652
Eligmodontia typus	—	—	—	AY277445	—	EU377610
Eliurus antsingy	—	—	—	—	—	HM223711
Eliurus carletoni	—	—	—	HM223845	—	HM223602
Eliurus ellermani	—	—	—	—	—	AF160573
Eliurus grandidieri	MF110364<br>AMNH 275218	—	—	MF097725<br>AMNH 275218	MF097847<br>AMNH 275218	KY753977<br>AMNH 275218
Eliurus majori	—	—	—	HM223872	—	AF160550
Eliurus minor	—	—	—	HM223876	—	AF160540
Eliurus myoxinus	MF110365<br>AMNH 275399	—	—	AY326085	MF097848<br>AMNH 275399	KY753978<br>AMNH 275399
Eliurus tanala	MF110366<br>USNM 448908	—	KC953258	KC953375	KC953497	KY753979<br>USNM 448908
Eliurus webbi	—	—	MF074875<br>MVZ 216998	MF097726<br>MVZ 216998	MF097849<br>MVZ 216998	KY753980<br>MVZ 216998
Ellobius talpinus	MF110367<br>RA	—	GQ142005	—	—	—
Ellobius tancrei	—	—	GQ142006	—	—	AF119270
Eolagurus luteus	—	—	GQ142004	—	—	—
Eospalax cansus	—	—	—	—	—	EF522072
Eospalax rufescens	—	—	—	—	—	FJ571147
Eospalax smithii	—	—	—	—	—	EF522064
Eothenomys chinensis	—	—	GQ374497	—	—	HM165437
Eothenomys custos	—	—	—	—	MF097850<br>UWBM 75296	AY426676
Eothenomys eva	—	—	—	—	—	HM165401
Eothenomys hintoni	—	—	—	—	—	HM165407
Eothenomys inez	—	—	—	—	—	HM165386

Eothenomys melanogaster mainland Asia	—	—	—	AY163583	—	HM165396
Eothenomys melanogaster Taiwan	MF110368 MVZ 180931	—	MF074876 MVZ 180931	MF097727 MVZ 180931	MF097851 MVZ 180931	KY753981 MVZ180931
Eothenomys miletus	MF110369 CAS 29092	—	—	—	MF097852 CAS 29092	HM165442
Eothenomys olitor	—	—	—	—	—	AY426688
Eothenomys proditor	—	—	—	—	—	KY753982 UWBM 75296
Eothenomys sp. 1	—	—	—	—	—	KY753983 CAS_27963
Eothenomys sp. 2	MF110370 UWBM 75247	—	—	MF097728 UWBM 75247	MF097853 UWBM 75247	KY753984 UWBM 75284
Eothenomys tarquinius	—	—	—	—	—	HM165398
Eremoryzomys polius	—	—	—	AY163624	—	EU579483
Euchoreutes naso	—	—	—	JF835099	—	—
Euneomys chinchilloides	MF110371 LB 018	KC953172	KC953259	MF097729 LB 018	KC953498	AY275115
Euneomys mordax	MF110372 MVZ 202625	—	—	—	—	KY753985 MVZ 202625
Euryoryzomys emmonsae	—	—	MF074877 MVZ 197524	MF097730 MVZ 197524	MF097854 MVZ 197524	AF251526
Euryoryzomys lamia	—	—	—	AY163619	—	—
Euryoryzomys macconnelli	—	—	—	AY163620	MF097855 MVZ 190450	KY753986 MVZ 190450
Euryoryzomys nitidus	MF110373 TK 14571	—	MF074878 TK 14571	MF097731 TK 14571	MF097856 TK 14571	KY753987 TK 14571
Euryoryzomys russatus	—	—	—	AY163625	—	KY753988 MVZ 192977
Geoxus valdivianus	MF110374 MVZ 154601	KC953173	KC953260	MF097732 MVZ 154601	KC953499	AY275116
Gerbilliscus afra	—	—	—	—	—	AJ430560
Gerbilliscus brantsii	—	—	—	—	—	AM409392
Gerbilliscus gambiana	—	—	—	—	—	AM409386
Gerbilliscus guineae	—	—	—	—	—	AM409379
Gerbilliscus kempi	—	—	—	—	—	AM409384
Gerbilliscus leucogaster	—	—	—	KR089043	—	AJ851260
Gerbilliscus nigricaudus	—	—	—	—	—	AM409378
Gerbilliscus robustus	KR088982	AY295005	AY294920	AY326113	KC953587	KR089018
Gerbilliscus validus	—	—	—	—	—	Z96044

Gerbillurus paeba	—	—	KC953261	KC953376	KC953500	KR089019
Gerbillurus setzeri	KR088983	—	KR089042	KR089044	KR089048	KR089020
Gerbillurus tytonis	KR088984	—	—	—	—	KR089021
Gerbillurus vallinus	KR088985	EU349643	AF332022	KC953377	AY294948	KR089022
Gerbillus gerbillus	KR088986	EU349700	DQ019049	EU349846	DQ023452	KR089023
Gerbillus latastei	—	—	—	—	—	GU356550
Gerbillus nanus	KR088987	—	KC953262	KC953378	KC953501	—
Gerbillus nigeriae	—	—	—	AM408333	—	AJ430555
Gerbillus tarabuli	—	—	—	—	—	GU356571
Golunda ellioti	—	—	AM910951	AM408332	—	AM408338
Grammomys cometes	—	—	—	—	—	EU275248
Grammomys dolichurus	—	—	EU349803	KC953379	KC953502	EU275253
Grammomys gazellae	—	—	—	AM408329	—	AM408345
Grammomys ibeanus	EU349614	KC953174	EU349801	KC953380	KC953503	KY753989<br>FMNH 151236
Grammomys macmillani	EU349615	KC953175	EU349802	EU349848	EU349888	KY753990<br>FMNH 155381
Grammomys surdaster	EU349616	—	—	—	—	KY753991<br>CM 102656
Graomys centralis	MF110375<br>PY 06	—	KC953263	KC953381	KC953504	KY753992<br>PY 07
Graomys chacoensis	—	—	—	—	—	FJ573153
Graomys domorum	MF110376<br>MSB 55291	—	—	MF097733<br>MSB 55291	—	AF159291
Graomys griseoflavus	MF110377<br>PY 03	KC953176	KC953264	AY277449	AY963181	KY753993<br>UP 110
Gymnuromys roberti	MF110378<br>USNM 449001	KC953177	AY294909	AY326087	KC953505	KY753994<br>USNM 449001
Habromys chinanteco	—	—	—	—	—	DQ861380
Habromys delicatulus	—	—	—	—	—	DQ861399
Habromys ixtlani	—	—	EF989832	EF989842	—	—
Habromys lepturus	MF110379<br>ROM 29849	KC953178	KC953265	EF989841	KC953506	KY753995<br>ROM 29849
Habromys lophurus	—	—	EF989745	EF989845	—	EF989944
Handleyomys alfaroi	MF110380<br>TK 136066	—	MF074879<br>TK 136066	MF097734<br>TK 136066	MF097857<br>TK 136066	KY753996<br>TK 136066
Handleyomys intectus	—	—	—	AY163584	—	EU579490
Handleyomys melanotis	MF110381	—	MF074880	MF097735	MF097858	—

	TK 101719		TK 101719	TK 101719	TK 101719	
Handleyomys rostratus	MF110382	—	MF074881	MF097736	MF097859	EU579493
	TK 113553		TK 113553	TK 113553	TK 113553	
Handleyomys saturatior	MF110383	—	MF074882	MF097737	MF097860	KY753997
	TK 113513		TK 113513	TK 113513	TK 113513	TK 113513
Hapalomys delacouri	—	—	—	MF097738	MF097861	KY753998
				CM 116069	CM 116069	CM 116069
Heimyscus fumosus	—	—	AM910953	DQ022397	—	AF518333
Hodomys alleni	MF110384	KC953179	KC953266	MF097739	MF097862	DQ179810
	2744 GOS			2744 GOS	2744 GOS	
Holochilus brasiliensis	—	—	—	EU273418	—	EU579495
Holochilus charcarius	—	—	MF074883	MF097740	MF097863	KY753999
			OMNHN 23525	OMNHN 23525	OMNHN 23525	OMNHN 23525
Holochilus sciureus	MF110385	—	KC953267	KC953382	KC953507	AF108697
	MVZ 193733					
Hybomys lunaris	—	—	—	—	—	DQ902763
Hybomys univittatus	MF110386	KC953181	DQ019059	KC953383	KC953508	KY754000
	CM 108044					CM 108044
Hydromys chrysogaster	EU349618	EU349699	EU349804	EU349849	EU349890	KY754001
						KU 160730
Hylaeamys acritus	MF110387	—	—	—	MF097864	AY940623
	USNM 597572				USNM 597572	
Hylaeamys laticeps	MF110388	—	MF074884	EU649050	MF097865	EU579498
	MVZ 197606		MVZ 197606		MVZ 197606	
Hylaeamys megacephalus	MF110389	—	MF074885	MF097741	MF097866	KY754002
	MVZ 197525		AMNH 257234	AMNH 257234	AMNH 25723	AMNH 25723
Hylaeamys perenensis	—	—	—	EU649051	—	KY754003
						MVZ 166676
Hylaeamys yunganus	MF110390	—	MF074886	MF097742	MF097867	KY754004
	TK 73788		TK 73788	TK 73788	TK 73788	TK 73788
Hylomyscus aeta	—	—	—	—	—	DQ078241
Hylomyscus alleni	MF110391	—	—	—	MF097868	AY057817
	CM 108090				CM 108090	
Hylomyscus baeri	—	—	—	—	—	DQ078244
Hylomyscus denniae	—	—	—	AY326088	—	DQ902733
Hylomyscus pamfi	—	—	—	—	—	HM013771
Hylomyscus parvus	MF110392	—	MF074887	DQ022399	DQ023479	CM 108105
	CM 108106		CM 108105			
Hylomyscus simus	—	—	—	—	—	DQ212188
Hylomyscus stella	—	—	AM910955	MF097743	MF097869	CM 102459

				CM 102459	CM 102459	
Hyomys goliath	MF110393<br>SAMA 42697	EU349679	EU349805	KC953384	MF097870<br>SAMA 42697	KY754005<br>SAMA 42697
Hypogeomys antimena	MF110394<br>MOR 149	—	AY294907	AY326089	KC953509	KY754006<br>MOR 149
Ichthyomys stolzmanni	—	—	—	MF097744<br>MUSM 25807	—	—
Irenomys tarsalis	—	KC953182	KC953268	MF097745<br>MVZ 155839	AY294962	U03534
Isthmomys pirrensis	MF110395<br>LSUMZ 25441	—	MF074888<br>LSUMZ 25441	MF097746<br>LSUMZ 25441	—	KY754007<br>LSUMZ 25441
Jaculus jaculus	—	—	AF332040	AM407907	JN414964	KY754008
Juliomys ossitenuis	—	—	—	—	—	AF108689
Juliomys pictipes	MF110396<br>MVZ 182079	KC953183	KC953269	KC953385	KC953510	AF108688
Juliomys rimofrons	—	—	—	—	—	AY029476
Juscelinomys huanchacae	MF110397<br>USNM 584508	—	—	AY277452	MF097871<br>USNM 584508	KY754009<br>USNM 584508
Kunsia tomentosus	MF110398<br>USNM 584516	—	—	KC953386	KC953511	AF108670
Lagurus lagurus	—	—	GQ142003	—	—	AF429818
Lasiopodomys brandtii	—	—	GQ374498	JF906134	—	BS0235
Lasiopodomys mandarinus	—	—	AM392396	AM919413	—	FJ986322
Leggadina forresti	DQ023437	EU349686	DQ019061	EU349850	DQ023468	EU349751
Lemmiscus curtatus	MF110399<br>MVZ 208364	—	—	—	—	KY754010<br>MVZ 208364
Lemmus lemmus	—	—	—	—	—	AY219145
Lemmus sibricus	MF110400<br>UWBM 39305	—	MF074889<br>UWBM 39305	MF097747<br>UWBM 39305	MF097872<br>UWBM 39305	KY754011<br>UWBM 39305
Lemniscomys barbarus	MF110401<br>CM 102462	KC953184	DQ019062	KC953387	DQ023461	KY754012<br>CM 102463
Lemniscomys bellieri	—	—	—	—	—	AF004586
Lemniscomys macculus	—	—	—	—	—	AF141208
Lemniscomys rosalia	—	—	—	DQ022390	—	AF141209
Lemniscomys striatus	—	—	AM910956	AM408321	—	AF141211
Lemniscomys zebra	—	—	—	—	—	AF141207
Lenoxus apicalis	MF110402<br>MVZ 171512	KC953185	KC953270	KC953388	KC953512	U03541
Leopoldamys edwardsi	MF110403<br>MVZ 186501	—	—	HM217687	MF097873<br>MVZ 186501	KY754013<br>MVZ 186501

Leopoldamys neilli	—	—	—	HM217697	—	HM217460
Leopoldamys sabanus	MF110404<br>RA Leopoldamys	KC953186	DQ019063	KC878208	KC953513	KC878030
Leporillus conditor	DQ070376	EU349692	EU349806	MF097748<br>SAMA 13335	MF097874<br>SAMA 13335	KY754014<br>SAMA 13335
Leptomys elegans	EU349619	EU349697	EU349807	MF097749<br>SAMA 45741	EU349893	KY754015<br>SAMA 45741
Limnomys bryophilus	—	—	GQ405380	DQ191508	—	DQ191479
Limnomys sibuanus	—	—	GQ405381	DQ191509	—	DQ191480
Lophiomys imhausi	KR088988	—	—	KC953389	KC953514	KR089025
Lophuromys aquilus	—	AY295006	AY294921	AY326091	AY294950	KY754016<br>FMNH 144777
Lophuromys brevicaudus	—	—	—	—	—	AY828232
Lophuromys chrysopus	—	—	—	—	—	AY828269
Lophuromys melanonyx	—	—	—	—	—	AY828235
Lophuromys sikapusi	—	—	KC953271	KC953390	KC953515	KR089026
Lophuromys woosnami	—	—	—	—	—	DQ902807
Lophuromys zena	—	—	KC953272	KC953391	KC953516	KR089027
Lorentzimys nouhuysi	—	EU349680	EU349808	KC953392	EU349894	KY754017<br>SAMA 42732
Loxodontomys micropus	MF110405<br>EUP 001	—	KC953273	AY277457	AY963183	AY275122
Lundomys molitor	—	—	—	AY163589	—	EU579501
Macrotarsomys bastardi	—	—	GQ272597	AY326092	—	EU497642
Macruromys major	EU349620	EU349678	MF074890<br>SAMA 43909	MF097750<br>SAMA 43909	EU349895	KY754018<br>SAMA 43909
Malacomys cansdalei	—	—	MF074891<br>CM 100502	—	MF097875<br>CM 100502	KY754019<br>CM 100502
Malacomys edwardsi	—	—	AM910958	—	—	—
Malacomys longipes	DQ023443	EU349656	DQ019064	DQ022393	DQ023474	KY754020<br>CM 108118
Malacothrix typica	MF110406<br>TM 39370	KC953186	AY294903	KC953393	KC953517	KY754021<br>TM 39370
Mallomys rothschildi	EU349621	EU349681	EU349810	MF097751<br>SAMA 47402	EU349896	KY754022<br>SAMA 47402
Mammelomys lanosus	EU349622	KC953188	EU349811	MF097752<br>SAMA 47208	EU349897	KY754023<br>SAMA 47208
Margaretamys elegans	MF110407<br>MSB 93144	—	KC953274	KC953394	KC953518	—
Mastacomys fuscus	DQ070378	EU349687	EU349812	MF097753	EU349898	KY754024

				SAMA 07354		SAMA 07354
Mastomys coucha	—	—	MF074892<br>CM 95050	MF097754<br>CM 95050	MF097876<br>CM 95050	KY754025<br>CM 95050
Mastomys erythroleucus	—	KC953189	AM910959	KC878210	KC953519	KY754026<br>SAMA 65697
Mastomys hildebrandti	—	AY295001	AY294916	—	KC953520	—
Mastomys huberti	—	—	—	—	—	AF141220
Mastomys kollmannspergeri	—	—	AM910961	—	—	HM635903
Mastomys natalensis	DQ070384	—	—	MF097755<br>FMNH 166943	—	KY754027<br>FMNH 166943
Mastomys pernanus	—	—	AM910960	DQ022403	—	AF518343
Mastomys verheyeni	—	—	—	DQ022402	—	—
Maxomys bartelsii	DQ023423	EU349666	DQ019066	EU349857	DQ023460	KY754028<br>SAMA 48063
Maxomys dollmani	—	—	—	—	—	KY754029<br>MSB 93274
Maxomys musschenbroekii	—	—	MF074893<br>MSB 93121	MF097756<br>MSB 93121	MF097877<br>MSB 93121	KY754030<br>MSB 93121
Maxomys rajah	—	—	—	KC878227	MF097878<br>MVZ 192210	KC878137
Maxomys surifer	—	KC953190	DQ019065	KC953396	—	HM217445
Maxomys whiteheadi	MF110408<br>LSUMZ M5122	—	MF074894<br>LSUMZ M5122	DQ191510	MF097879<br>LSUMZ M5122	EU292150
Megadontomys cryophilus	—	—	—	—	—	DQ861373
Megadontomys thomasi	—	—	EF989750	EF989850	—	AY195795
Melanomys caliginosus	—	—	—	—	—	EU340020
Melanomys cf. idoneus	MF110409<br>LSUMZ 25552	—	MF074895<br>LSUMZ 25552	MF097757<br>LSUMZ 25552	MF097880<br>LSUMZ 25552	EU340023
Melanomys chrysomelas	MF110410<br>USNM 464387	KC953191	MF074896<br>USNM 464387	EU649053	MF097881<br>USNM 464387	KY754031<br>USNM 464387
Melanomys columbianus	—	—	—	—	—	EU340022
Melasmothrix naso	EU349624	—	EU349815	KC953398	—	KY754032<br>SAMA 65765
Melomys burtoni	JN114321	—	—	—	—	—
Melomys capensis	JN114335	—	—	—	—	—
Melomys cervinipes	EU349625	—	—	KC953399	EU349901	—
Melomys leucogaster	JN114339	—	—	—	—	—
Melomys lutillus	JN114319	—	—	—	—	—
Melomys rubicola	JN114337	—	—	—	—	—

Melomys rufescens	EU349626	EU349690	EU349816	MF097758 EU349860	EU349902	KY754033 SAMA 43071
Meriones chengi	—	—	—	—	—	AB381900
Meriones crassus	KR088990	—	—	—	—	KR089028
Meriones libycus	—	—	—	—	—	KR089029
Meriones meridianus	—	—	—	—	—	AJ851268
Meriones persicus	KR088991	—	—	—	—	KR089030
Meriones rex	—	—	—	—	—	AJ851265
Meriones shawi	—	AF332048	AF332021	KC953400	AY294947	—
Meriones tamariscinus	—	—	—	—	—	AB381904
Meriones tristrami	KR088992	—	—	—	—	KR089032
Meriones unguiculatus	KR088993	—	FN984754	AY326095	—	KR089031
Mesembriomys gouldii	DQ070382	EU349693	EU349817	MF097759 SAMA 07412	EU349903	KY754034 SAMA 07412
Mesocricetus auratus	MF110411 RA Cricetinae	AY295013	AF540632	AY163591	AY294955	KY754035
Mesocricetus brandti	—	—	—	—	—	AM904628
Mesocricetus newtoni	—	—	—	—	—	AJ973381
Mesocricetus raddei	—	—	—	—	—	AJ973382
Micaelamys namaquensis	MF110412 RA 12	EU349649	AY294914	AM408330	AY294941	KY754036 RA 12
Microakodontomys transitorius	—	—	—	EU649054	—	—
Micromys minutus	EU349627	EU349664	EU349818	EU349862	EU349904	AB201995
Microryzomys minutus	MF110413 MVZ 173975	—	KC953276	AY163592	KC953522	AF108698
Microtus agrestis	MF110414 MVZ 159217	—	AM910792	AM919427	MF097882 MVZ 159217	AY167174
Microtus arvalis	—	—	AM392386	AM919416	—	AM991024
Microtus californicus	MF110415 MVZ 207423	—	KC953277	KC953401	KC953523	KY754037 MVZ 207423
Microtus chrotorrhinus	—	—	AM392383	AM919403	—	AF163893
Microtus daghestanicus	—	—	GQ142009	—	—	—
Microtus duodecimcostatus	—	—	AM392400	—	—	—
Microtus fortis	—	—	GQ374494	JF906129	—	FJ986308
Microtus gregalis	—	—	GQ142007	—	—	—
Microtus guentheri	—	—	AM392397	AM919420	—	AY513805

Microtus kikuchii	MF110416<br>MVZ 201001	—	AM392385	AM919410	—	KY754038<br>MVZ 201001
Microtus limnophilus	—	—	—	AM919426	—	AM910620
Microtus longicaudus	MF110417<br>MVZ 196704	—	AM392379	AM919414	MF097883<br>MVZ 196704	KY754039<br>MVZ 196704
Microtus majori	—	—	AM910796	AM919409	—	—
Microtus maximowiczii	—	—	—	—	—	FJ986312
Microtus middendorffi	—	—	AM392390	AM919419	—	FJ986314
Microtus mongolicus	—	—	—	—	—	FJ986310
Microtus montanus	MF110418<br>FMNH 145964	—	KC953278	KC953402	KC953524	KY754040<br>FMNH 145962
Microtus montebelli	—	—	AM910793	AM919421	—	—
Microtus ochrogaster	MF110419<br>TK 123149	—	MF074897<br>TK 123149	MF097760<br>TK 123149	MF097884<br>TK 123149	KY754041<br>TK 123149
Microtus oeconomus	MF110420<br>LSUMZ 311219	—	AM392388	AM919418	—	AY305142
Microtus pennsylvanicus	—	AY295009	AF540633	AM919415	AY241463	AF119279
Microtus pinetorum	MF110421<br>OMNHN 37823	—	MF074898<br>OMNHN 37823	MF097761<br>OMNHN 37823	MF097885<br>OMNHN 37823	KY754042<br>OMNHN 37823
Microtus richardsoni	—	—	AM392387	AM919404	—	AF163905
Microtus sachalinensis	—	—	—	—	—	FJ986318
Microtus schelkovnikovi	—	—	AM910794	AM919408	—	KY754043<br>MVZ 179136
Microtus socialis	MF110422<br>MVZ 198799	—	FM162073	FM162055	—	AY513830
Microtus thomasi	—	—	AM910797	AM919422	—	AY513844
Microtus transcaspicus	—	—	AM910795	AM919405	—	—
Microtus xanthognathus	—	—	—	—	—	KY754044<br>MVZ 149059
Millardia kathleenae	EU349628	—	MF074899	KC953403	EU349905	KY754045
Millardia meltada	—	—	AM910962	AM408322	—	AF141221
Monticolomys koopmani	—	—	GQ272598	AY326096	—	AF160580
Monticolomys sp.	MF110423<br>AMNH 275217	—	—	—	MF097886<br>AMNH 275217	KY754046<br>AMNH 275217
Mus baoulei	—	—	—	—	—	EU603998
Mus booduga	—	—	—	AB125796	AB125818	AB125760
Mus bufo	—	—	—	—	—	DQ789905
Mus caroli	MF110424<br>AMNH 275650	—	—	MF097762<br>AMNH 275650	MF097887<br>AMNH 275650	KY754047<br>AMNH 275650
Mus cervicolor	—	—	—	AB125799	AB125823	AY057811

Mus cookii	MF110425<br>USNM 583802	—	KC953279	KC953404	MF097888<br>USNM 583802	AY057813
Mus crociduroides	—	—	—	AJ698894	—	AJ698878
Mus famulus	MF110426<br>RA	—	—	AJ698884	—	AJ698872
Mus fragilicauda	—	—	—	AB125812	—	AB125779
Mus gratus	—	—	—	—	—	AY263613
Mus haussa	—	—	—	AJ698891	—	AJ875071
Mus indutus	—	—	—	AJ698892	—	AJ698874
Mus lepidoides	—	—	—	AB262417	—	AB262414
Mus macedonicus	—	—	—	AB125805	—	AB125770
Mus mattheyi	—	—	—	AJ698889	—	AJ698876
Mus minutoides	—	—	—	AJ875086	—	AJ875076
Mus musculoides	—	—	—	—	—	AJ698875
Mus musculus	M99054	EU349657	M33324	NM015745	AY241462	AF520621
Mus nitidulus	—	—	—	AB262425	—	AB269818
Mus pahari	EU349629	—	KC953280	EU349864	EU349906	EU349767
Mus platythrix	—	—	—	AJ698895	—	—
Mus saxicola	—	—	—	—	—	AY057815
Mus setulosus	—	—	—	AJ875088	—	GU830865
Mus sorella	—	—	—	—	—	DQ789904
Mus spicilegus	MF110427<br>MVZ 192264	—	MF074900<br>MVZ 192264	AJ698882	MF097889<br>MVZ 192264	KY754048<br>MVZ 192264
Mus spretus	MF110428<br>MVZ 155909	—	—	AJ698883	MF097890<br>MVZ 155909	AY224678
Mus tenellus	—	—	—	—	—	DQ789903
Mus terricolor	—	—	—	AB125810	AB125837	AY193770
Mylomys dybowskii	—	—	AM910965	EU292146	—	AF141212
Myodes andersoni	—	—	AM392391	—	—	AB037281
Myodes californicus	MF110429<br>MVZ 208823	—	MF074901<br>MVZ 208823	MF097763<br>MVZ 208823	MF097891<br>MVZ 208823	KY754049<br>MVZ 208823
Myodes centralis	—	—	—	—	—	DQ845185
Myodes gapperi	—	AY295010	MF074902<br>FMNH 145956	AY326080	AY294952	KY754050<br>FMNH 145956
Myodes glareolus	MF110430<br>MVZ 179108	JF930108	JF930127	AM919401	—	AF119272
Myodes regulus	—	—	—	—	—	DQ138116
Myodes rex	—	—	—	—	—	AB031582
Myodes rufocanus	—	AY133336	GQ142002	—	—	FJ792791
Myodes rutilus	—	JF930113	JF930129	MF097764	—	SAF09021

				TK 123027		
Myodes smithii	—	—	—	—	—	AB104508
Myomyscus brockmani	—	—	AM910966	DQ022407	—	—
Myomyscus verreauxii	—	—	MF074903<br>CM 95080	MF097765<br>CM 95080	MF097892<br>CM 95080	KY754051<br>CM 95080
Myomyscus yemeni	—	—	AM910968	DQ022409	—	AF518357
Myopus schisticolor	—	—	GQ142001	—	—	AF119263
Myospalax aspalax	MF110431<br>MSB 100533	KC953192	KC953281	AY326097	KC953525	KY754052<br>MSB 100533
Myospalax psilurus	—	—	—	—	—	AF326271
Myotomys sloggetti	—	—	—	—	—	AF492724
Myotomys unisulcatus	—	—	—	—	—	AF492729
Mystromys albicaudatus	—	—	GQ272600	AY163594	—	AF160606
Napaeozapus insignis	MF110432<br>H 678	AF540634	KC953282	AY326098	KC953526	KY754053<br>H 678
Neacomys dubosti	—	—	—	—	—	FM210781
Neacomys guianae	—	—	—	—	—	FM210778
Neacomys minutus	MF110433<br>MVZ 193750	—	KC953283	MF097766<br>MVZ 193750	KC953527	KY754054<br>MVZ 193750
Neacomys musseri	MF110434<br>MVZ 193763	—	MF074904<br>MVZ 193763	MF097767<br>MVZ 193763	MF097893<br>MVZ 193763	KY754055<br>MVZ 193763
Neacomys paracou	—	—	—	—	—	FM210765
Neacomys spinosus	MF110435<br>MVZ 155015	—	KC953284	KC953406	KC953528	U58391
Necromys amoenus	MF110436<br>MVZ 172877	KC953193	KC953285	MF097768<br>MVZ 172877	KC953529	AY273911
Necromys lactens	—	—	—	—	—	EF531646
Necromys lasiurus	—	—	—	—	MF097894<br>MVZ 197603	U03528
Necromys lenguarum	MF110437<br>USNM 584521	—	—	—	—	EF531676
Necromys obscurus	—	—	—	—	—	DQ683183
Necromys temchuki	—	—	—	AY277461	—	AY273914
Necromys urichi Amazonas	—	—	—	—	—	U03549
Necromys urichi Bolivar	—	—	—	AY277463	—	AY273918
Nectomys apicalis	MF110438<br>MVZ 166700	—	KC953286	KC953407	KC953530	EU340013
Nectomys squamipes	MF110439<br>MVZ 192933	KC953194	KC953287	MF097769<br>MVZ 192933	KC953531	EU340012
Neodon irene	—	—	AY294924	AM919412	AY241464	GU908312

Neodon juldaschi	—	—	—	—	—	EF599113
Neodon sikimensis	—	—	GQ374496	AY163593	—	—
Neofiber alleni	MF110440<br>USNM 568085	—	—	AM919425	MF097895<br>USNM 568085	AM910618
Neotoma albigula	MF110441<br>MVZ 197066	—	MF074905<br>MVZ 197066	MF097770<br>MVZ 197066	—	AF186814
Neotoma bryanti	MF110442<br>MVZ 195972	—	KC953288	KC953408	KC953532	KY754056<br>MVZ 195972
Neotoma cinera	—	—	—	KC953409	KC953533	KY754057<br>MVZ 207659
Neotoma devia	MF110443<br>MVZ 197117	—	—	KC953410	KC953534	KY754058<br>MVZ 197117
Neotoma floridana	MF110444<br>OK 107	KC953195	AY294959	KC953411	AY294959	KY754059<br>OK 107
Neotoma fuscipes	—	—	—	—	—	DQ179823
Neotoma goldmani	—	—	—	—	—	DQ179827
Neotoma isthmica	—	—	—	—	—	DQ179828
Neotoma lepida	MF110445<br>TK 119266	—	MF074906<br>TK 119266	MF097771<br>TK 119266	MF097896<br>TK 119266	KY754060<br>TK 119266
Neotoma leucodon	—	—	—	—	—	DQ179839
Neotoma macrotis	MF110446<br>MVZ 196550	—	—	—	—	KY754061<br>MVZ 196550
Neotoma magister	—	—	—	—	—	DQ179856
Neotoma mexicana	MF110447<br>TK 122944	—	MF074907<br>TK 122944	MF097772<br>TK 122944	MF097897<br>TK 122944	KY754062<br>TK 122944
Neotoma micropus	MF110448<br>TK 116316	—	MF074908<br>TK 116316	MF097773<br>TK 116316	MF097898<br>TK 116316	KY754063<br>TK 116316
Neotoma picta	—	—	—	—	—	DQ179851
Neotoma stephensi	MF110449<br>MVZ 197173	—	—	—	MF097899<br>MVZ 197173	KY754064<br>MVZ 197173
Neotomodon alstoni	MF110450<br>MSB 418171	KC953196	KC953289	KC953412	KC953535	KY754065<br>MSB 418171
Neotomys ebriosus	MF110451<br>JSB 506	—	KC953290	KC953413	KC953536	KY754066<br>JSB 506
Nephelomys albigularis	MF110452<br>LSUMZ 26987	—	—	AY163614	MF097900<br>LSUMZ 26987	EU579505
Nephelomys keaysi	—	—	KC953291	KC953414	KC953537	KY754067<br>MVZ 173987
Nephelomys levipes	MF110453<br>MVZ 166680	—	—	KC953415	KC953538	KY754068<br>MVZ 166680

Nesokia indica	—	—	—	—	—	AF160605
Nesomys audiberti	—	—	—	—	MF097901<br>TRA 231	KY754069<br>TRA 231
Nesomys rufus	MF110454<br>USNM 448955	KC953197	KC953292	AY326099	KC953539	KY754070<br>USNM 448955
Nesoryzomys fernandinae	—	—	—	EU649058	—	AF108700
Nesoryzomys indefessus	MF110455<br>MVZ 145384	—	—	AY163600	—	—
Nesoryzomys swarthi	—	—	—	AY163601	—	EU340014
Niviventer andersoni	MF110456<br>USNM 574357	—	—	—	—	EF053003
Niviventer brahma	—	—	—	—	—	EF053010
Niviventer confucianus	MF110457<br>USNM 574365	—	KC953293	KC953416	KC953540	JF714942
Niviventer coninga	—	—	—	—	—	EF053024
Niviventer cremoriventer	MF110458<br>F 35796	KC953198	DQ019067	KC953417	KC953541	KY754071<br>F 35796
Niviventer culteratus	MF110459<br>MVZ 180686	KC953199	DQ019068	KC953418	DQ023458	GU479941
Niviventer eha	—	—	—	—	—	EF053018
Niviventer excelsior	MF110460<br>USNM 574373	—	EQ405386	KC953419	MF097902<br>USNM 574373	GU479941
Niviventer fulvescens	—	—	JN009859	HM217657	—	—
Niviventer langbianis	MF110461<br>AMNH 272447	—	—	HM217671	MF097903<br>AMNH 272447	GU457008
Niviventer niviventer	—	—	—	AM408323	—	—
Niviventer rapit	—	—	—	—	—	DQ191483
Niviventer tenaster	—	—	—	MF097774<br>AMNH 274355	MF097904<br>AMNH 274355	KY754072<br>AMNH 274355
Notiomys edwardsii	MF110462<br>MVZ 163067	KC953200	KC953294	KC953420	KC953542	U03537
Notomys alexis	—	—	—	—	—	AY176318
Notomys fuscus	DQ070379	—	KC953295	EU360811	EU349907	KY754073<br>SAMA 34070
Nyctomys sumichrasti	—	KC953201	KC953296	KC953421	MF097905<br>MSB 45815	KY754074<br>MSB 45815
Ochrotomys nuttalli	MF110463<br>CM 106431	KC953202	KC953297	KC953422	KC953543	KY754075<br>MSB 53299
Oecomys auyantepui	—	—	—	—	—	AJ496304
Oecomys bicolor	—	—	KC953298	KC953423	KC953544	AF108699

Oecomys catherinae	MF110464 MVZ 200982	—	—	AY163605	—	EU579507
Oecomys concolor	—	—	—	AY163606	—	—
Oecomys mamorae	—	—	MF074909 MVZ 197692	MF097775 MVZ 197692	—	EU579509
Oecomys paricola	MF110465 MVZ 198000	—	MF074910 MVZ 198000	—	—	KY754076 MVZ 198000
Oecomys rex	—	—	—	—	—	AJ496314
Oecomys roberti	—	—	—	—	—	KY754077 MVZ 200930
Oecomys rutilus	—	—	—	—	—	AJ496312
Oecomys superans	—	KC953203	KC953300	AY277464	KC953546	KY754078 MVZ 155008
Oecomys tapajinus	—	—	—	—	—	EU579508
Oecomys trinitatis	—	—	MF074911 MVZ 200953	MF097776 MVZ 200953	MF097906 MVZ 200953	U58390
Oenomys hypoxanthus	MF110466 CM 102549	EU349654	DQ019069	KC953425	DQ023464	KY754079 CM 102548
Oligoryzomys andinus	—	—	—	—	—	L37400
Oligoryzomys chacoensis	—	—	MF074912 OMNHN 34273	MF097777 OMNHN 34273	MF097907 OMNHN 34273	L37387
Oligoryzomys destructor	MF110467 OMNHN 34310	—	—	EU649061	—	KY754080 OMNHN 34310
Oligoryzomys eliurus	MF110468 MVZ 183083	—	—	MF097778 MVZ 183083	MF097908 MVZ 183083	EU192163
Oligoryzomys flavescens	—	—	—	DQ826030	—	DQ826015
Oligoryzomys fornesi	—	—	—	DQ826033	—	EU192158
Oligoryzomys fulvescens	MF110469 MVZ 155316	KC953204	—	AY163611	KC953547	—
Oligoryzomys longicaudatus	MF110470 MVZ 155842	—	KC953302	KC953426	KC953548	GQ282541
Oligoryzomys magellanicus	—	—	—	—	—	AY275705
Oligoryzomys messorius	—	—	—	DQ826032	—	DQ826024
Oligoryzomys microtis	MF110471 MVZ 190440	—	—	MF097779 MVZ 190440	KC953549	FJ374766
Oligoryzomys moojeni	—	—	—	DQ826031	—	DQ826019
Oligoryzomys nigripes	—	—	—	DQ826029	—	EU192161
Oligoryzomys stramineus	—	—	—	AY163613	—	DQ826026
Oligoryzomys vegetus	—	—	—	EU649067	—	L37386
Ondatra zibethicus	KR088994	AY295011	AY294925	KC953427	AY294953	KY754081

Onychomys arenicola	—	—	EF989755	EF989856	—	AY195793
Onychomys leucogaster	—	—	KC953303	EF989860	KC953550	AY195794
Onychomys torridus	MF110472<br>MVZ 206851	—	—	EF989861	—	KY754082<br>ASNHC 4066
Oreoryzomys balneator	—	—	—	EU649068	—	EU258534
Oryzomys azuerensis	—	—	—	—	—	EU074669
Oryzomys couesi	MF110473<br>CM 41027	AF332043	MF074913<br>CM 41027	AY163618	—	KY754083<br>CM 41027
Oryzomys mexicanus	—	—	—	—	—	EU074646
Oryzomys palustris	MF110474<br>MSB 74956	KC953205	KC953304	AY163623	KC953551	KY754084<br>MSB 74956
Osgoodomys banderanus	—	—	EF989757	EF989858	—	KY754085<br>TK 11796
Otomys anchietae	—	—	GQ405388	AY326101	—	—
Otomys angoniensis	—	SAMA 65830	MF074914<br>SAMA 65830	AM408325	MF097909<br>SAMA 65830	KY754086<br>SAMA 65830
Otomys denti	—	—	KC953305	KC953428	—	EU874434
Otomys irroratus	—	—	—	—	—	KY754087<br>CAS 28393
Otomys jacksoni	—	—	—	—	—	AF492727
Otomys lacustris	—	—	—	—	—	EU874445
Otomys laminatus	—	—	—	—	—	FJ619560
Otomys maximus	—	—	—	—	—	AF492723
Otomys occidentalis	—	—	—	—	—	JF795981
Otomys saundersiae	—	—	—	—	—	FJ619559
Otomys tropicalis	—	—	—	—	—	KY754088<br>FMNH 161144
Otomys typus	—	—	—	—	—	EU874432
Ototylomys phyllotis	MF110475<br>F 35529	AY295018	AY294932	KC953429	KC953553	KY754089<br>F 35529
Oxymycterus amazonicus	MF110476<br>USNM 549815	—	MF074915<br>USNM 549815	—	MF097910<br>USNM 549815	AF454765
Oxymycterus dasytrichus	MF110477<br>MVZ 183127	—	—	—	MF097911<br>MVZ 183127	KY754090<br>MVZ 183127
Oxymycterus delator	MF110478<br>MVZ 197931	—	MF074916<br>MVZ 197931	MF097780<br>MVZ 197931	MF097912<br>MVZ 197931	U03525
Oxymycterus hiska	MF110479<br>MVZ 171518	—	KC953306	KC953430	KC953554	U03542
Oxymycterus hispidus	MF110480<br>MVZ 193008	—	—	—	—	KY754091<br>MVZ 193008

Oxymycterus josei	MF110481<br>MVZ 193035	—	—	—	MF097913<br>MVZ 193035	AF175289
Oxymycterus nasutus	MF110482<br>MVZ 182701	KC953206	KC953307	KC953431	KC953555	KY754092<br>MVZ 182701
Oxymycterus paramensis Argentina	MF110483<br>OMNHN 34968	—	MF074917<br>OMNHN 34968	MF097781<br>OMNHN 34968	MF097914<br>OMNHN 34968	DQ518261
Oxymycterus paramensis Peru	—	—	—	—	—	U03536
Oxymycterus quaestor	—	—	—	—	—	AF454772
Oxymycterus rufus	MF110484<br>OMNHN 30079	—	MF074918<br>OMNHN 30079	MF097782<br>OMNHN 30079	MF097915<br>OMNHN 30079	AY275126
Oxymycterus wayku	—	—	—	—	—	DQ518262
Pachyuromys duprasi	—	—	—	—	—	AJ851274
Parahydromys asper	EU349631	EU349698	EU349820	MF097783<br>SAMA 45798	EU349910	KY754093<br>SAMA 45798
Paramelomys levipes	EU349632	EU349689	EU349821	EU349867	MF097916<br>KU 160736	KY754094<br>KU 160736
Paramelomys platyops	MF110485<br>MVZ 141054	—	—	—	—	KY754095<br>MVZ 141054
Paramelomys rubex	JN114347	—	—	—	—	—
Parotomys brantsii	MF110486<br>H 656	EU349646	AY294912	KC953432	MF097917<br>H 656	KY754096<br>H 656
Paruromys dominator	EU349634	EU349669	EU349822	KC953433	—	KY754097<br>SAMA 65763
Pearsonomys annectens	—	—	—	AY851749	—	AF108672
Pelomys fallax	—	—	—	DQ022391	—	DQ022382
Peromyscus attwateri	MF110487<br>OMNHN 33377	—	MF074919<br>OMNHN 33377	MF097784<br>OMNHN 33377	MF097918<br>OMNHN 33377	KY754098<br>OMNHN 33377
Peromyscus aztecus	MF110488<br>LSUMZ 25106	—	KC953308	KC953434	KC953556	EF989968
Peromyscus boylii	MF110489<br>MVZ 216481	—	KC953309	KC953435	KC953557	AF155386
Peromyscus californicus	MF110490<br>MVZ 199654	—	EF989772	EF989873	—	KY754099<br>MVZ 199654
Peromyscus cf. hylocetes	—	—	—	—	—	KY754100<br>LSUMZ 25106
Peromyscus cf. spicilegus	—	—	—	—	—	KY754101<br>CM 103724
Peromyscus crinitus	MF110491<br>MVZ 217321	—	KC953310	KC953436	KC953558	KY754102<br>MVZ 217321
Peromyscus difficilis	MF110492	—	MF074920	—	MF097919	KY754103

	LSUMZ 36247		LSUMZ 36247		LSUMZ 36247	LSUMZ 36247
Peromyscus eremicus	MF110493	—	MF074921	EF989877	MF097920	KY754104
	LSUMZ 34364		LSUMZ 34364		LSUMZ 34364	LSUMZ 34364
Peromyscus fraterculus	MF110494	—	KC953311	KC953437	KC953559	KY754105
	USNM 569216					USNM 569216
Peromyscus furvus	—	—	—	—	—	AF271027
Peromyscus gossypinus	MF110495	—	—	—	MF097921	DQ973102
	LSUMZ 26782				LSUMZ 26782	
Peromyscus keeni	—	—	—	—	—	X89787
Peromyscus leucopus	MF110496	AY295014	AY294927	EF989880	MF097922	KY754106
	OK 14				OK 015	OK 014
Peromyscus levipes	MF110497	—	EF989782	EF989882	—	KY754107
	MVZ 159526					MVZ 159526
Peromyscus maniculatus California	—	—	—	—	MF097923	KY754108
					MVZ 200760	MVZ 200760
Peromyscus maniculatus Northern range	—	—	EF989783	AY163630	—	DQ385808
Peromyscus mayensis	—	—	EF989787	EF989888	—	EF989987
Peromyscus melanophrys	—	—	—	EF989890	—	—
Peromyscus melanotis	—	—	EF989790	—	—	X89791
Peromyscus mexicanus Costa Rica	—	—	EF989793	EF989894	—	EF989993
Peromyscus mexicanus Veracruz	—	—	—	—	—	EF028174
Peromyscus nudipes	—	—	EF989792	EF989893	—	—
Peromyscus polionotus	—	—	EF989795	EF989896	—	EF989995
Peromyscus polius	—	—	—	—	—	AF155403
Peromyscus truei	MF110498	—	—	AY277413	—	KY754109
	MVZ 197293					MVZ 197293
Peromyscus winkelmanni	—	—	—	—	—	U89983
Petromyscus collinus	—	—	—	DQ191517	—	AF160600
Petromyscus monticularis	MF110499	AY294999	AY294906	—	MF097924	KY754110
	RA 14				RA 14	RA 14
Phaiomys leucurus	—	—	AM392394	AM919400	—	AM392371
Phenacomys intermedius	MF110500	—	AM392377	KC953438	MF097925	KY754111
	MVZ 201914				MVZ 201914	MVZ 201914
Phloeomys cumingi	—	—	—	AY326103	—	DQ191484
Phloeomys pallidus	DQ023451	EU349644	DQ019070	KC8878237	DQ023480	—
Phodopus campbelli	—	—	—	—	—	AF119278
Phodopus roborovskii	—	—	—	—	—	EF025539
Phodopus sungorus	—	AY295012	AF540640	KC953439	AY294954	KY754112
Phyllotis alisosiensis	—	—	—	—	—	GQ119626

Phyllotis amicus	MF110501<br>UNSM 10787	—	—	—	AY963207	AY956708
Phyllotis andium	MF110502<br>ORB 125	—	KC953312	MF097785<br>UNSM 10750	AY963204	AY956706
Phyllotis anitae	—	—	—	—	—	AY627298
Phyllotis bonariensis	MF110503<br>SA 02	—	—	—	AY963250	AY956731
Phyllotis caprinus	—	—	MF074922<br>OMNHN 30082	MF097786<br>OMNHN 30082	—	KY754113<br>OMNHN 30082
Phyllotis darwini	MF110504<br>MSB 69977	—	AF332023	—	AY963212	AY956729
Phyllotis gerbillus	MF110505<br>ORB 58	—	—	—	AY963206	AY956713
Phyllotis haggardi	—	—	MF074923<br>TK 104756	MF097787<br>TK 104756	MF097926<br>TK 104756	KY754114<br>TK 104756
Phyllotis limatus	MF110506<br>ORB 34	—	—	—	—	AY956740
Phyllotis magister	MF110507<br>FMNH 107691	—	—	—	AY963234	AY956721
Phyllotis osilae	MF110508<br>MSB 67272	KC953207	KC953313	KC953440	KC953560	AY956711
Phyllotis xanthopygus posticalis	—	—	—	—	—	KY754115<br>ORB_92
Phyllotis xanthopygus xanthopygus	KR088995	KC953208	KC953314	AY163632	KC953561	U86833
Podomys floridanus	—	—	EF989778	EF989879	—	DQ973110
Pogonomys loriae	EU349635	EU349683	EU349823	KC953441	EU349912	EU349776
Pogonomys macrourus	EU349636	EU349684	EU349824	MF097788<br>SAMA 43144	EU349913	KY754116<br>SAMA 43144
Pogonomys sylvestris	—	—	GQ405389	GQ405365	—	—
Praomys daltoni	—	—	AM910972	DQ022406	—	AF518348
Praomys degraaffi	—	—	KC953315	KC953442	KC953562	KY754117<br>FMNH 138046
Praomys delectorum	—	—	—	AY326104	—	JQ735742
Praomys derooi	—	—	—	DQ022405	—	AF518350
Praomys hartwigi	—	—	JF284245	—	—	AF518367
Praomys jacksoni	MF110509<br>CM 102583	EU349663	DQ019071	KC953443	DQ023477	KY754118<br>CM 102584
Praomys misonnei	MF110510<br>FMNH 149576	—	KC953316	KC953444	KC953563	KY754119<br>FMNH 149576
Praomys morio	—	—	JF284240	—	—	—

Praomys obscurus	—	—	JF284248	—	—	—
Praomys petteri	—	—	JF284249	—	—	AF518368
Praomys rostratus	—	—	JF284239	—	—	GU397446
Praomys tullbergi	DQ023448	EU349662	DQ019072	DQ022413	DQ023478	KY754120<br>CM 108199
Praomys verschureni	—	—	—	DQ022394	—	AF518373
Proedromys bedfordi	—	—	GQ374485	JF906130	—	—
Proedromys liangshanensis	—	—	GQ374492	JF906133	—	—
Prometheomys schaposchnikowi	—	—	AM392395	AM919406	—	AM392372
Psammomys obesus	KR088996	—	MF074924<br>TK 40892	FN357290	MF097927<br>TK 40892	KR089035
Psammomys vexillaris	—	—	—	—	—	AY934541
Pseudohydromys ellermani	EU349623	EU349695	EU349814	MF097789<br>SAMA 43920	MF097928<br>SAMA 43920	KY754121<br>SAMA 43920
Pseudomys australis	—	EU349688	DQ019073	EU349870	DQ023469	AM910936
Pseudomys chapmani	—	—	—	—	—	AY176320
Pseudomys desertor	—	—	—	—	—	AY176326
Pseudomys hermannsburgensis	—	—	—	—	—	AY176321
Pseudomys shortridgei	—	—	—	—	—	FJ946452
Pseudoryzomys simplex	MF110511<br>MSB 80490	—	KC953317	AY163633	KC953564	KY754122<br>MSB 80490
Punomys kofordi	MF110512<br>VPT 1890	KC953209	KC953318	KC953445	KC953565	KY754123<br>VPT 1890
Rattus andamanensis	—	—	—	HM217641	MF097929<br>AMNH 272324	KY754124<br>AMNH 272324
Rattus argentiventer	—	—	—	HM217602	MF097930<br>MSB 93171	KY754125<br>MSB 93171
Rattus colletti	—	—	—	HQ334598	—	—
Rattus everetti	—	—	—	DQ191513	—	DQ191485
Rattus exulans	MF110513<br>NK 80010	—	DQ019074	KC953446	DQ023455	KY754126<br>NK 80010
Rattus facetus	—	—	—	—	—	KY754127<br>MSB 93164
Rattus fuscipes	—	—	—	HQ334610	—	EF186435
Rattus giluwensis	—	HQ334419	—	HQ334606	HQ334673	—
Rattus hoffmanni	—	—	—	—	—	EF186441
Rattus leucopus	MF110514<br>KU 160771	EU349672	EU349825	HQ334621	EU349914	KY754128<br>KU 160770
Rattus losea	—	—	—	—	—	HM031715
Rattus lutreolus	—	—	—	—	—	GU570671

Rattus niobe	—	—	—	HQ334580	—	—
Rattus nitidus	—	—	—	HM217711	—	HM217479
Rattus norvegicus	DQ023418	EU349671	X16726	AB033709	AY294938	KY754129
Rattus novaeguineae	MF110515 SAMA 46853	KC953210	KC953319	KC953447	KC953566	KY754130 SAMA 46853
Rattus praetor	MF110516 USNM 580077	—	GQ405392	KC953448	KC953567	DQ191487
Rattus rattus	—	—	AM910976	HM217606	HQ334643	—
Rattus satarae	—	—	—	HM217749	—	HM217739
Rattus sordidus	—	HQ334411	—	HQ334599	HQ334691	EF186480
Rattus steini	—	—	—	HQ334588	—	—
Rattus tanezumi	—	—	—	HM217694	—	FR775851
Rattus tiomanicus	KR088997	—	KC953320	KC953449	KC953568	HM217391
Rattus tunneyi	—	—	—	HQ334612	—	EF186518
Rattus verecundus	MF110517 SAMA 43425	KC953211	KC953321	MF097790 SAMA 43425	KC953569	KY754131 SAMA 43425
Rattus villosissimus	DQ070372	EU349673	EU349826	HQ334576	MF097931 SAMA 00549	KY754132 SAMA 00549
Reithrodon auritus Entre Rios	—	—	—	—	—	AY275129
Reithrodon auritus Rio Negro	MF110518 MVZ 182707	KC953212	AY294930	AY277472	MF097932 MVZ 182707	KY754133 MVZ 182707
Reithrodontomys brevirostris	—	—	EF989817	EF989918	—	EF990017
Reithrodontomys creper	MF110519 ROM 113346	—	KC953322	KC953450	KC953570	AY859429
Reithrodontomys dariensis	—	—	EF989815	EF989916	—	EF990015
Reithrodontomys fulvescens	MF110520 OK 326	AY295015	AY294928	EF989904	AY294958	KY754134 OK 326
Reithrodontomys gracilis	MF110521 JM Rg13	—	EF989807	EF989905	KC953571	AY859432
Reithrodontomys humulis	MF110522 OMNHN 36692	—	MF074925 OMNHN 36692	MF097791 OMNHN 36692	MF097933 OMNHN 36692	KY754135 OMNHN 36692
Reithrodontomys megalotis	HQ269526	—	KC953323	AY277414	KC953572	KY754136 ASHNC 2136
Reithrodontomys mexicanus	HQ269527	—	EF989805	EF989906	—	KY754137 JM K
Reithrodontomys microdon	MF110523 ROM 98300	—	EF989814	EF989915	—	KY754138 ROM 98300
Reithrodontomys spectabilis	MF110524 JM L	—	EF989822	EF989923	—	AY859462
Reithrodontomys sumichrasti	MF110525	—	EF989824	EF989924	MF097934	KY754139

	ROM 98384				ROM 98384	ROM 98384
Rhabdomys dilectus	—	—	—	—	—	FR837633
Rhabdomys pumilio	MF110526	EU349650	AY294913	EU349871	AY294940	KY754140
	RA 23					RA 23
Rhagomys longilingua	MF110527	—	MF074926	MF097792	—	KY754141
	FMNH 175218		FMNH 175218	FMNH 175218		FMNH 175218
Rhagomys rufescens	—	—	—	—	—	AY206770
Rheomys raptor	MF110528	—	—	AY163635	MF097935	KY754142
	KU 159017				KU 159017	KU 159017
Rheomys thomasi	—	—	KC960491	KC953451		—
					KR089049	
Rhipidomys cariri	—	—	—	—	—	HM594666
Rhipidomys emilae	—	—	—	—	—	AF108682
Rhipidomys gardneri	—	—	—	—	—	U03550
Rhipidomys ipukensis	—	—	—	—	—	HM594633
Rhipidomys itoan	—	—	—	—	—	HM594658
Rhipidomys leucodactylus	MF110529	—	MF074927	MF097793	MF097936	HQ634183
	MVZ 190590		MVZ 190590	MVZ 190590	MVZ 190590	
Rhipidomys macconnelli	MF110530	KC953213	KC953324	AY277474	KC953573	AF108681
	MVZ 160083					
Rhipidomys macrurus	MF110531	—	—	—	—	KY754143
	MVZ 197543					MVZ 197543
Rhipidomys masticalis	MF110532	KC953214	AY294929	KC953452	AY294961	AF108684
	MVZ 193037					
Rhipidomys nitela	MF110533	—	MF074928	MF097794	MF097937	HM594665
	MVZ 197548		MVZ 197548	MVZ 197548	MVZ 197548	
Rhipidomys sp. 1	—	—	—	—	—	AF108683
Rhipidomys tribei	—	—	—	—	—	HM594663
Rhipidomys venezuelae	MF110534	—	—	—	MF097938	KY754144
	AMNH 24857				AMNH 24857	AMNH 24857
Rhipidomys venustus	MF110535	—	—	MF097795	MF097939	KY754145
	AMNH 24865			AMNH 24865	AMNH 24865	AMNH 24865
Rhipidomys wetzeli	—	—	—	—	—	AF108680
Rhizomys pruinosus	—	—	AY294899	MF097796	KC953574	KY754146
				MVZ 176525		MVZ 176525
Rhombomys opimus	—	—	—	—	—	AJ430556
Rhynchomys isarogensis	AY324462	EU349677	DQ019075	KC953453	AY294944	EU349784
Saccostomus campestris	MF110536	KC953215	KC953325	AY326109	KC953575	KY754147
	SAMA 65838					SAMA 65838
Salinomys delicatus	MF110537	—	MF074929	MF097797	MF097940	EU377608

	OMNHN 23602		OMNHN 23602	OMNHN 23602	OMNHN 23602	
Scapteromys aquaticus	—	—	—	AY277476	—	AY275132
Scapteromys tumidus	MF110538	—	KC953326	AY277477	KC953576	KY754148
	MVZ 183267					MVZ 183267
Scolomys juruaense	MF110539	—	KC953327	KC953454	KC953577	—
	MVZ 183165					
Scolomys melanops	—	—	—	—	—	AF527419
Scolomys ucayalensis	—	—	MF074930	MF097798	MF097941	EU579518
			AMNH 272721	AMNH 272721	AMNH 272721	
Scotinomys teguina	MF110540	KC953216	KC953328	MF097799	KC953578	AF108705
	JM B			JM B		
Scotinomys xerampelinus	MF110541	—	—	MF097800	MF097942	KY754149
	LSUMZ 25166			JM N	LSUMZ 25166	ROM_97311
Sekeetamys calurus	KR088998	—	—	—	—	AJ851276
Sicista concolor	—	—	—	JF835107	—	—
Sicista tianshanica	MF110542	—	KC953329	AF297288	KC953579	KY754150
	RA Sicistinae					
Sigmodon alleni Michoacan	—	—	—	—	—	AF155425
Sigmodon alleni Oaxaca	MF110543	—	MF074931	MF097801	MF097943	KY754151
	TK 150546		TK 150546	TK 150546	TK 150546	TK 150546
Sigmodon alstoni	MF110544	KC953217	KC953330	KC953455	KC953580	KY754152
	AMCC 112960					AMCC 112960
Sigmodon arizonae	MF110545	KC953218	KC953331	EU635700	KC953581	KY754153
	MSB 54086					MSB 54086
Sigmodon fulviventer	MF110546	—	MF074932	MF097802	MF097944	KY754154
	TK 72392		TK 72392	TK 72392	TK 72392	TK 72392
Sigmodon hirsutus	—	—	MF074933	MF097803	MF097945	AY517528
			TK 119145	TK 119145	TK 119145	
Sigmodon hispidus	KR088999	AY295016	AF540641	AY277479	AY241465	AF425200
Sigmodon leucotis	—	—	—	EU635712	—	AF293401
Sigmodon mascotensis	MF110547	—	MF074934	MF097804	MF097946	AF155424
	TK 150550		TK 150550	TK 150550	TK 150550	
Sigmodon ochrognathus	MF110548	—	MF074935	MF097805	MF097947	AF155422
	TK 48160		TK 48160	TK 48160	TK 48160	
Sigmodon peruanus	—	—	—	EU635719	—	AF293395
Sigmodon toltecus	MF110549	—	MF074936	MF097806	MF097948	AF425228
	TK 136997		TK 136997	TK 136997	TK 136997	
Sigmodontomys alfari	MF110550	KC953219	KC953332	AY163641	KC953582	KY754155
	USNM 449895					USNM 575662
Solomys ponceleti	JN114358	—	—	—	—	—

Solomys salebrosus	EU349638	EU349691	EU349827	EU349872	EU349917	EU349785
Sooretamys angouya	MF110551<br>MVZ 182806	—	KC953333	AY163616	KC953583	KY754156<br>MVZ 192961
Soricomys kalinga	—	—	—	—	—	JQ898059
Soricomys leonardocoi	—	—	—	JQ898077	—	JQ898062
Soricomys montanus	—	—	—	JQ898076	—	JQ898066
Soricomys musseri	—	—	—	JQ898075	—	JQ898071
Spalax carmeli	—	—	—	—	—	JN571137
Spalax ehrenbergi	—	JN414208	AY294898	KC953405	AB303250	KY754157<br>H 150
Spalax galili	—	—	—	—	—	JN571129
Spalax judaei	—	—	—	—	—	JN571135
Spalax leucodon	—	—	—	—	—	FJ656299
Spalax nehringi	—	—	—	—	—	GQ374526
Spalax zemni	—	—	—	SZU48589	—	—
Srilankamys ohiensis	—	—	JN009860	—	—	—
Steatomys krebsi	—	KC953220	KC953334	KC953457	KC953584	KY754158<br>CM 6328
Steatomys parvus	—	—	GQ272602	AY326110	—	AF160598
Stenocephalemys albipes	—	—	AM910977	DQ022404	—	AF518347
Stenocephalemys albocaudata	—	—	AM910978	DQ022414	—	AF518370
Stenocephalemys griseicauda	—	—	—	—	—	AF518371
Stochomys longicaudatus	DQ023429	EU349652	DQ019076	KC953458	KC953585	KY754159<br>CM 108122
Sundamys muelleri	DQ023420	EU349668	DQ019077	AY326111	DQ023456	KY754160<br>MVZ 192334
Synaptomys borealis	—	—	MF074937<br>MSB 158056	MF097807<br>MSB 158056	—	KY754161<br>MSB 158056
Synaptomys cooperi	MF110552<br>USNM 568611	KC953221	KC953335	KC953459	KC953586	KY754162<br>USNM 568611
Tachyoryctes akoliae	MF110553<br>RA	—	—	—	MF097949	KY754163
Tachyoryctes audax	MF110554<br>FMNH 190310	—	MF074938<br>FMNH 190310	—	—	KY754164<br>FMNH 190310
Tachyoryctes ruandae	—	—	MF074939<br>FMNH 157817	—	—	—
Tachyoryctes splendens	—	KC953222	AY294900	AY326112	—	AF160602
Tapecomys primus	—	—	—	—	—	AF159288
Tapecomys wolffsohni	MF110555<br>MSB 63364	KC953223	KC953336	KC953460	AY963184	AY956698

Tarsomys apoensis	—	—	GQ405395	DQ191516	—	DQ191491
Tateomys rhinogradoides	MF110556<br>SAMA 65766	—	—	—	—	—
Tatera indica	—	—	—	—	—	AJ430563
Taterillus arenarius	—	—	—	FN357288	—	AJ851261
Taterillus emini	KR08900	KC953224	DQ019050	KC953461	DQ023453	—
Taterillus gracilis	—	—	—	—	—	AJ851263
Taterillus pygargus	—	—	—	—	—	AJ851262
Thallomys loringi	—	—	—	—	—	DQ381928
Thallomys nigricauda	MF110557<br>CAS 28624	—	—	—	—	DQ381925
Thallomys paedulcus	—	—	—	—	—	DQ381926
Thallomys sp.	—	—	MF074940<br>CAS 28611	—	MF097950<br>CAS 28611	—
Thalpomys cerradensis	—	—	—	AY277481	—	AY273915
Thalpomys lasiotis	—	—	—	—	—	AY310347
Thaptomys nigrita	MF110558<br>MVZ 183040	KC953225	KC953337	AY277482	KC953588	KY754165<br>MVZ 183040
Thomasomys andersoni	—	—	—	—	—	DQ914643
Thomasomys aureus	MF110559<br>MVZ 170076	KC953226	KC953338	KC953462	KC953589	U03540
Thomasomys australis	—	—	—	—	—	DQ914645
Thomasomys baeops	MF110560<br>AMNH 268146	—	—	MF097808<br>AMNH 268146	MF097951<br>AMNH 268146	DQ914654
Thomasomys caudivarius	MF110561<br>MSB 70717	KC953227	KC953339	KC953463	KC953590	DQ914648
Thomasomys cinnameus	—	—	—	—	—	DQ914646
Thomasomys daphne LAC	—	—	—	—	—	KY754166<br>LAC 104
Thomasomys daphne Puno	MF110562<br>MVZ 171501	—	MF074941<br>MVZ 171501	MF097809<br>MVZ 171501	—	KY754167<br>MVZ 171501
Thomasomys erro	—	—	—	—	—	EU579476
Thomasomys gracilis	—	—	—	MF097810<br>MVZ 166669	—	AF108674
Thomasomys ischyrus	—	—	—	AY277484	—	AF108675
Thomasomys kalinowskii	—	—	—	—	—	AF108678
Thomasomys ladewi	—	—	—	—	—	DQ914647
Thomasomys macrotis	MF110563<br>LSUMZ 27287	—	—	—	—	KY754168<br>LSUMZ 27287
Thomasomys notatus	MF110564	—	KC953340	KC953464	KC953591	AF108676

	MVZ 166706					
Thomasomys onkiro	MF110565<br>USNM 582124	—	MF074942<br>USNM 582124	—	MF097952<br>USNM 582124	KY754169<br>USNM 582124
Thomasomys oreas	—	—	—	—	—	DQ914651
Thomasomys paramorum	MF110566<br>TK 104764	—	MF074943<br>TK 104764	MF097811<br>TK 104764	MF097953<br>TK 104764	—
Thomasomys sp. 1	—	—	—	—	—	AF108677
Thomasomys sp. 2	—	—	—	—	—	DQ914653
Tokudaia muenninki	—	—	—	AB548697	AB548692	AB548693
Tokudaia osimensis	EU349640	EU349659	EU349828	EU349874	EU349918	AB033703
Tokudaia tokunoshimensis	—	—	—	AB548698	—	AB548693
Tonkinomys daovantieni	—	—	MF074944<br>AMNH 275712	—	MF097954<br>AMNH 275712	KY754170<br>AMNH 275712
Transandinomys bolivaris	—	—	—	EU649073	—	EU579513
Transandinomys bombycinus	—	—	—	MF097812<br>LSUMZ 26352	MF097955<br>LSUMZ 26352	KY754171<br>LSUMZ 26352
Transandinomys talamancae	—	KC953228	KC953341	KC953465	KC953592	KY754172<br>MSB 91815
Tscherskia triton	—	—	—	—	MF097956<br>UWBM 77335	KY754173<br>UWBM 77335
Tylomys nudicaudus<br>El Salvador	—	CN 103590	AY294933	AY163643	KC953593	KY754174<br>F 35721
Tylomys nudicaudus Guatemala	—	—	—	—	—	DQ179812
Tylomys watsoni	MF110567<br>USNM 464885	—	—	KC953466	KC953594	KY754175<br>USNM 464885
Typhlomys cinereus	—	—	GQ272603	GQ272606	—	—
Uranomys ruddi	DQ023417	EU349642	DQ019051	EU360812	DQ023454	HM635858
Uromys caudimaculatus	DQ023439	—	DQ019079	EU349875	MF097957<br>MVZ 193100	KY754176<br>MVZ 193100
Uromys hadrourus	JN114353	—	—	—	—	—
Vandeleuria oleracea	EU349641	EU349655	EU349829	MF097813	EU349919	KY754177
Voalavo gymnocaudus	—	—	GQ272604	AY326114	—	AF160596
Volemys millicens	—	—	GQ374480	JF906135	—	—
Volemys musseri	MF110568<br>UWBM 75266	—	GQ374483	JF906128	—	KY754178<br>UWBM 75266
Wiedomys pyrrhorhinos	MF110569<br>UFMG 1912	—	KC953342	AY277485	KC953595	AY275134
Xenomys nelsoni	MF110570<br>TK 28546	KC953229	KC953343	MF097814<br>TK 28546	MF097958<br>TK 28546	KY754179<br>TK 28546
Xeromys myoides	DQ070380	EU349696	EU349830	EU349877	EU349920	EU349790

Zapus princeps	MF110571<br>FMNH 163053	—	AF332041	AF297287	MF097959<br>FMNH 145965	KY754180<br>FMNH 163053
Zelotomys hildegardeae	MF110572<br>CM 102659	EU349661	DQ019080	DQ022396	DQ023476	KY754181<br>CM 102661
Zygodontomys brevicauda	MF110573<br>AMCC 113004	KC953230	KC953344	JF906128	KC953596	KY754182<br>AMCC 113004
Zygodontomys cherriei	—	—	—	AY163646	—	EU579520
Zygodontomys microtinus	—	—	—	—	—	EU652764
Zygodontomys sp. 1	—	—	—	—	—	EU652750
Zygodontomys sp. 2	—	—	—	—	—	EU652758
Zyzomys argurus	DQ070381		EU349685	MF097815<br>SAMA 07908	EU349831	KY754183<br>SAMA 07908

GenBank sequences indicated with GenBank accession numbers (e.g., "EU652764") that lack spaces between letters and numbers. Newly determined sequences are referenced by GenBank accessions and followed by museum vouchers (e.g., "FMNH 145965") or collector numbers, that have spaces between acronyms and numbers. Abbreviations for museums and collectors are as follows: The Ambrose Monell Collection for Molecular and Microbial Research, American Museum of Natural History, AMCC; Angelo State Natural History Collection, ASNHC; California Academy of Sciences, CAS; Carnegie Museum of Natural History, CM; Universidad Nacional Autónoma de México, CNMA; Field Museum of Natural History, FMNH; Louisiana State University Museum of Natural History, LSUMZ; Museum of Southwestern Biology, MSB; Museum of Vertebrate Zoology, Berkeley, MVZ; Sam Noble Oklahoma Museum of Natural History, OMNHN; Royal Ontario Museum, ROM; Southwestern Australian Museum, SAMA; Texas Tech University, TK; Universidade Federal de Minas Gerais, UFMG; University of Kansas Natural History Museum and Biodiversity Research Center, UK; Museo de Historia Natural, Peru, UNSM; United States National Museum, USNM; University of Washington, Burke Museum, UWBM. CN is the tissue accession prefix for the Royal Ontario Museum. NK is the tissue accession prefix for the Museum of Southwestern Biology. AK refers to specimens catalogued at the Texas Cooperative Wildlife Collection. JSB refers to uncatalogued specimens housed at Texas Tech, and collector numbers H refer to uncatalogued specimens housed in the Texas Cooperative Wildlife Collections and collected by members of the laboratory of Dr. Rodney Honeycutt. JM refers to specimens collected by J. Miller (ROM). ORB refers to collections of Oswaldo Ramirez-Baca. RA and F refer to uncatalogued specimens from the collections of Ronald Adkins. SP refers to specimens catalogued at Carnegie Museum of Natural History. MOR refers to specimens collected from the Omaha Henry Doorly Zoo and Aquarium. PY refers to specimens collected by Ulysses Pardiñas. VPT refers to specimens collected by Victor Pacheco Torres. EPU and LB refers to specimens housed at Centro Nacional Patagonico, Argentina. RH and MAW are accessions associated with the University of Memphis. All available collecting locality information for new sequences can be found on GenBank.
