# Supplementary figures and images for: Muroid rodent phylogenetics: 900-species tree reveals increasing diversification rates
Source: PLoS One. 2017 Aug 16;12(8):e0183070. doi: 10.1371/journal.pone.0183070 (PMC5559066; doi:10.1371/journal.pone.0183070)

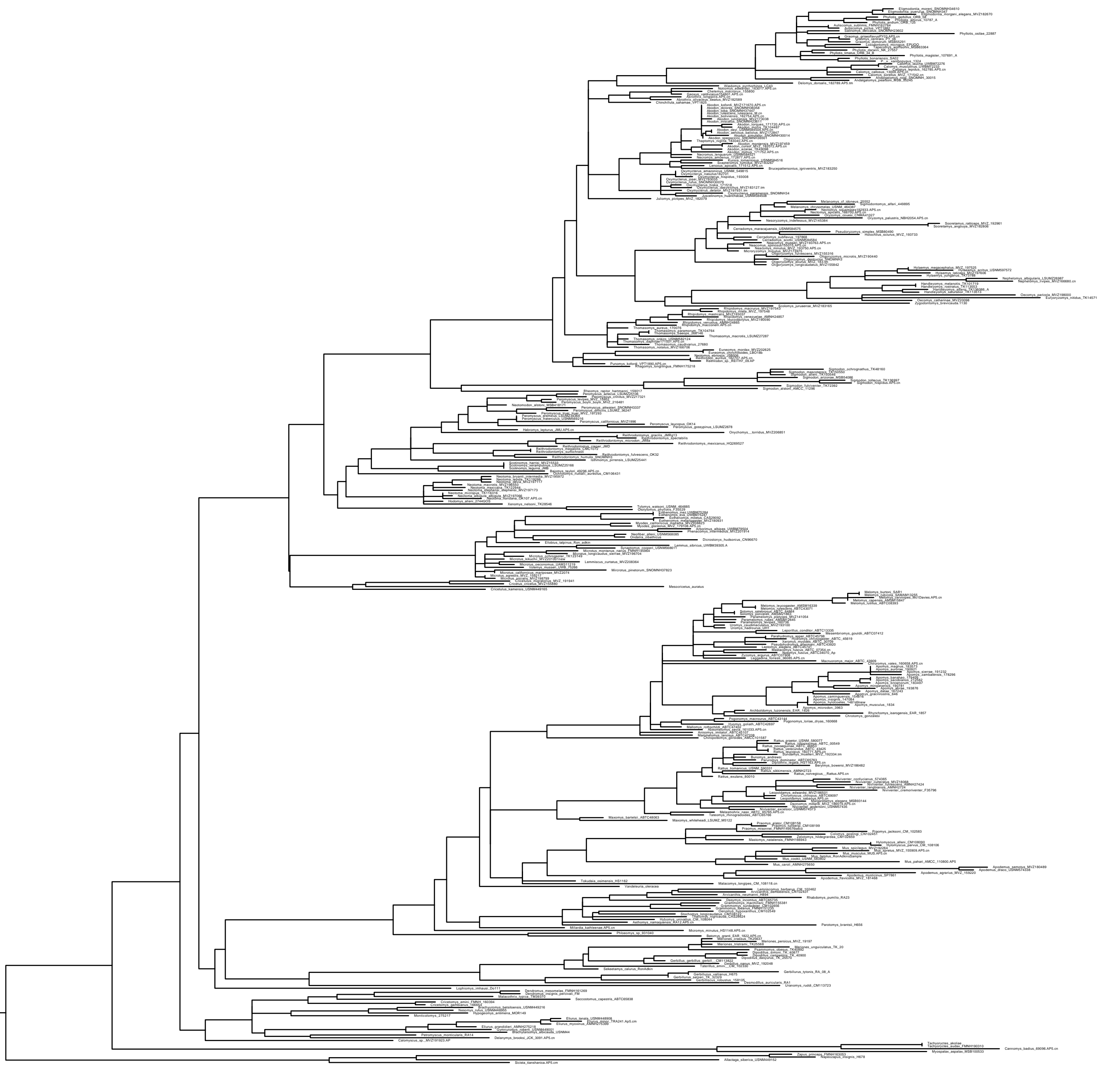

Supplement: S1 Fig — (PDF) [file pone.0183070.s002.pdf]

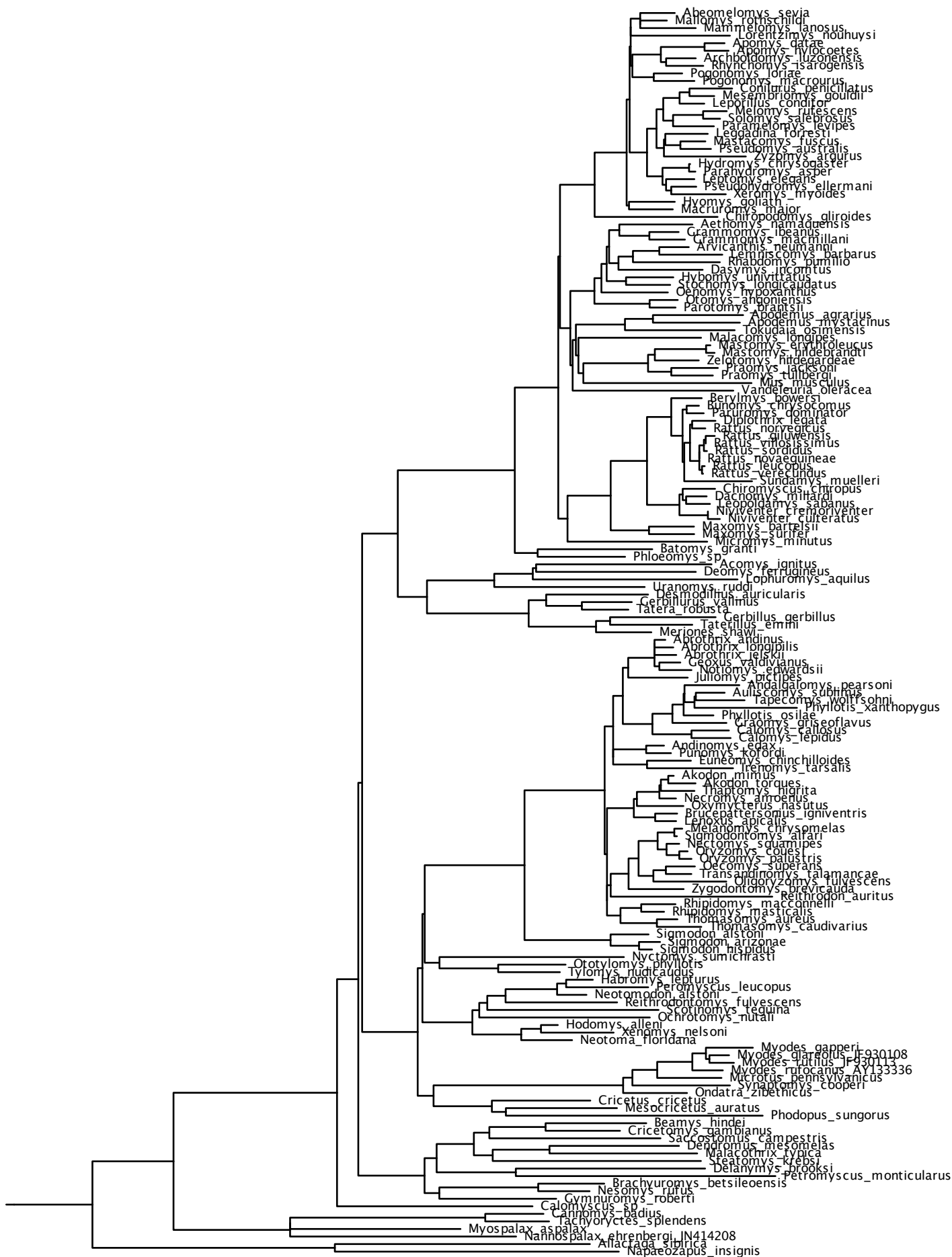

0.04

Supplement: S2 Fig — (PDF) [file pone.0183070.s003.pdf]

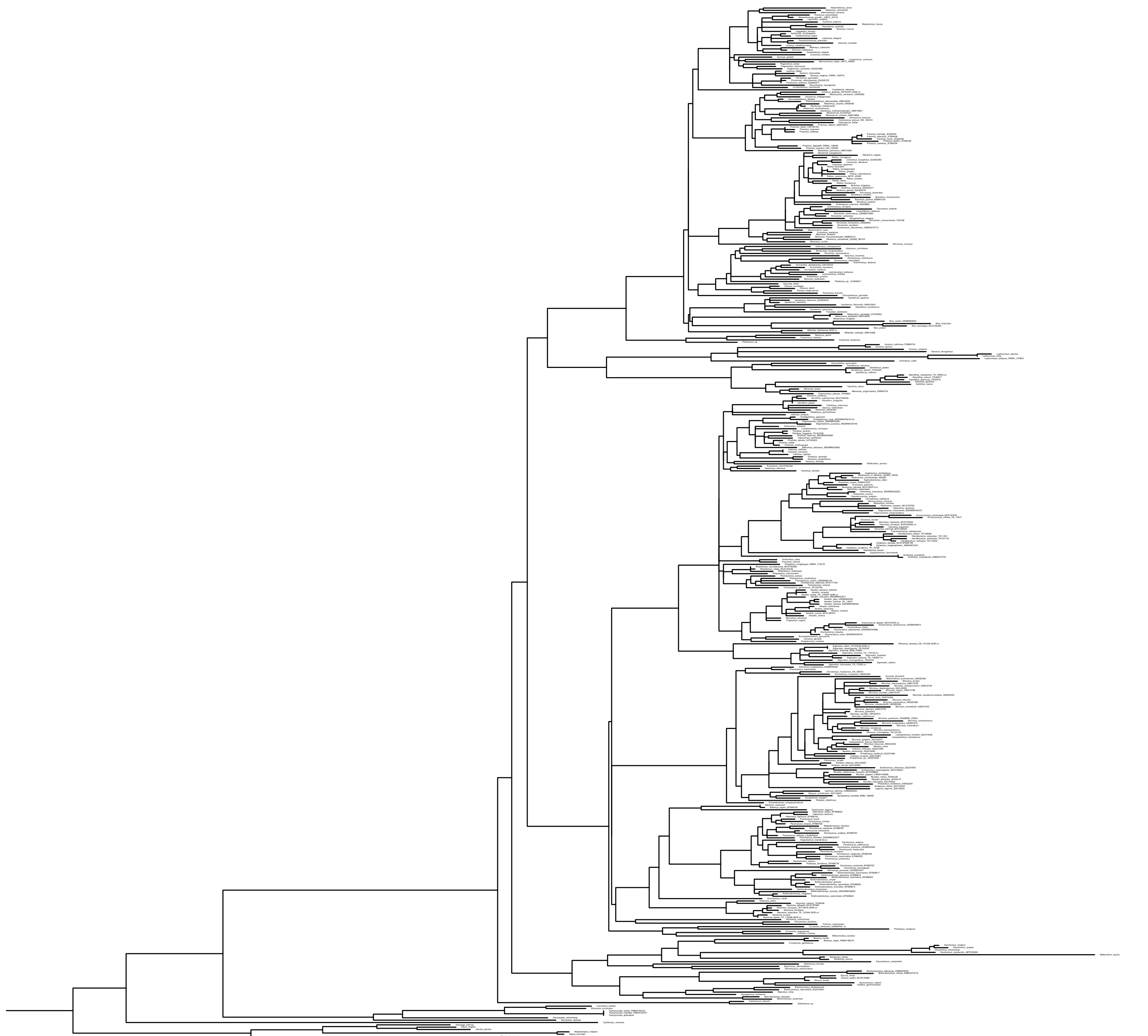

0.04

Supplement: S3 Fig — (PDF) [file pone.0183070.s004.pdf]

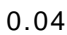

Supplement: S4 Fig — (PDF) [file pone.0183070.s005.pdf]

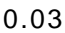

Supplement: S5 Fig — (PDF) [file pone.0183070.s006.pdf]

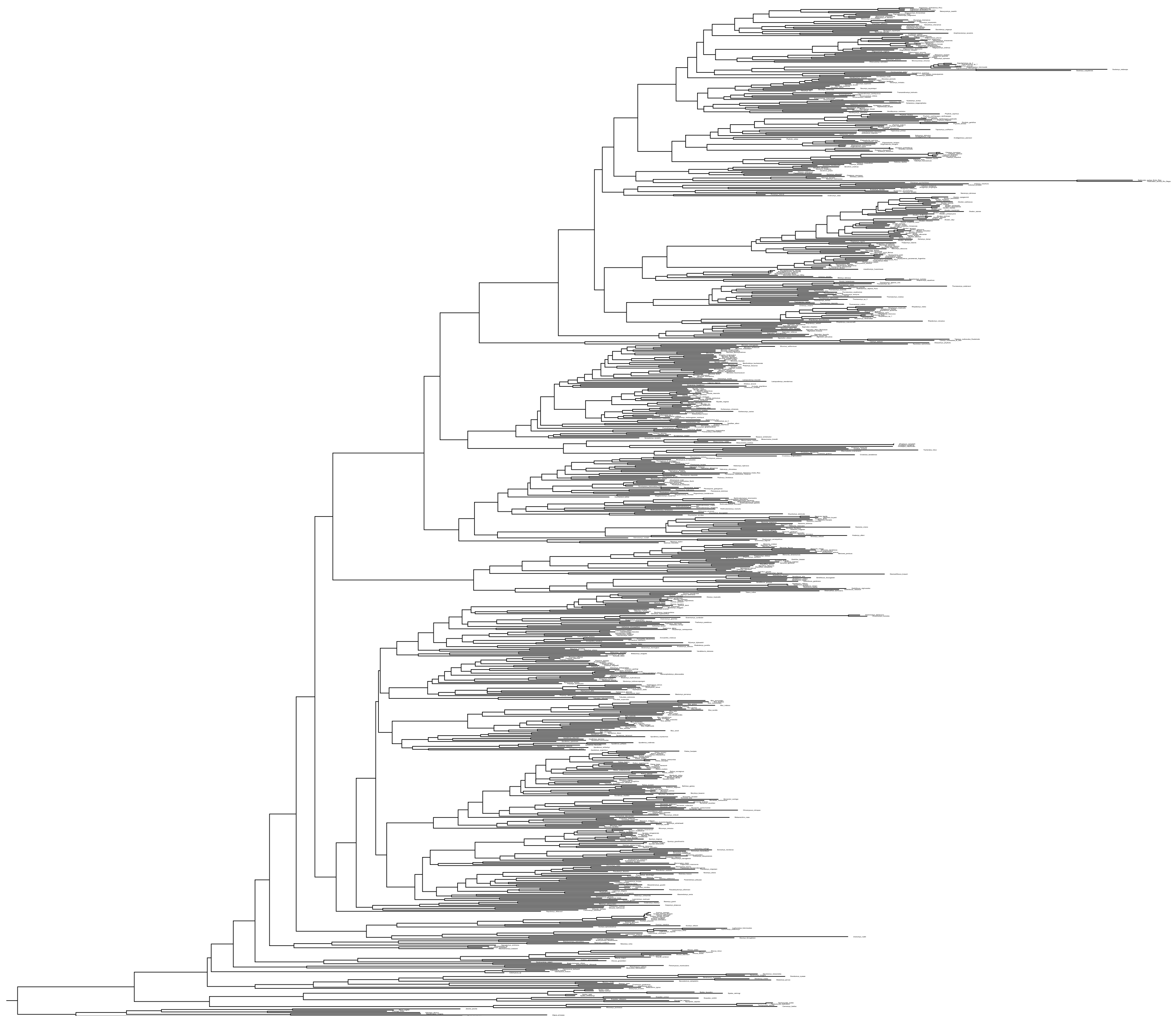

0.3

Supplement: S6 Fig — (PDF) [file pone.0183070.s007.pdf]

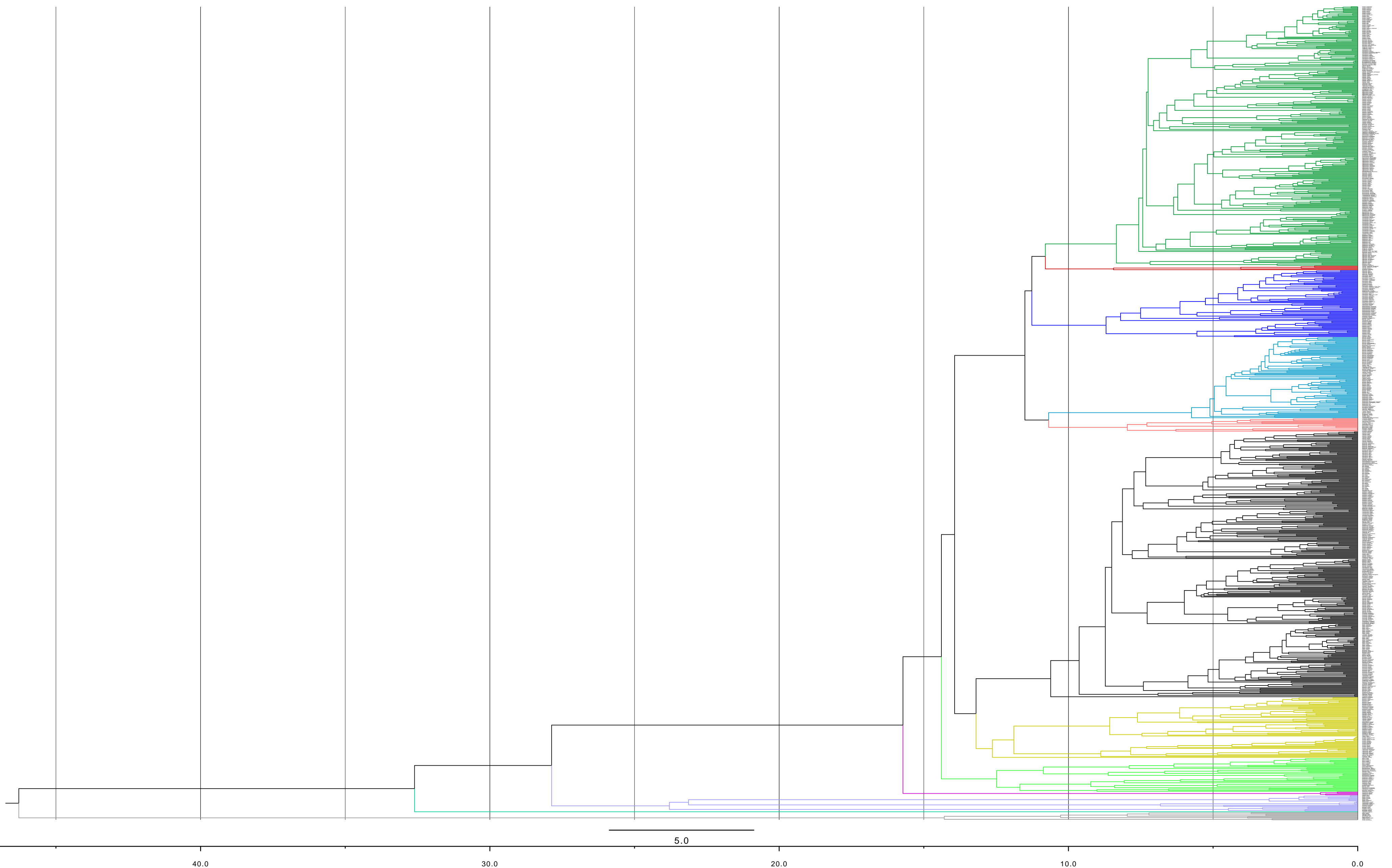

Supplement: S7 Fig — (PDF) [file pone.0183070.s008.pdf]
